# Molecular Basis for ATP-Hydrolysis-Driven DNA Translocation by the CMG Helicase of the Eukaryotic Replisome

**DOI:** 10.1016/j.celrep.2019.07.104

**Published:** 2019-09-03

**Authors:** Patrik Eickhoff, Hazal B. Kose, Fabrizio Martino, Tatjana Petojevic, Ferdos Abid Ali, Julia Locke, Nele Tamberg, Andrea Nans, James M. Berger, Michael R. Botchan, Hasan Yardimci, Alessandro Costa

**Affiliations:** 1 Macromolecular Machines Laboratory, The Francis Crick Institute, London NW1 1AT, UK; 2 Single Molecule Imaging of Genome Duplication and Maintenance Laboratory, The Francis Crick Institute, London NW1 1AT, UK; 3 Department of Molecular and Cell Biology, University of California, Berkeley, Berkeley, CA 94720, USA; 4 Institute of Technology, University of Tartu, Tartu 50411, Estonia; 5 Structural Biology Science Technology Platform, The Francis Crick Institute, 1 Midland Road, London NW1 1AT, UK; 6 Department of Biophysics and Biophysical Chemistry, Johns Hopkins University School of Medicine, Baltimore, MD 21205, USA

**Keywords:** DNA replication, cryo-EM, AAA+ ATPase, helicase, molecular motor, DNA unwinding

## Abstract

In the eukaryotic replisome, DNA unwinding by the Cdc45-MCM-Go-Ichi-Ni-San (GINS) (CMG) helicase requires a hexameric ring-shaped ATPase named minichromosome maintenance (MCM), which spools single-stranded DNA through its central channel. Not all six ATPase sites are required for unwinding; however, the helicase mechanism is unknown. We imaged ATP-hydrolysis-driven translocation of the CMG using cryo-electron microscopy (cryo-EM) and found that the six MCM subunits engage DNA using four neighboring protomers at a time, with ATP binding promoting DNA engagement. Morphing between different helicase states leads us to suggest a non-symmetric hand-over-hand rotary mechanism, explaining the asymmetric requirements of ATPase function around the MCM ring of the CMG. By imaging of a higher-order replisome assembly, we find that the Mrc1-Csm3-Tof1 fork-stabilization complex strengthens the interaction between parental duplex DNA and the CMG at the fork, which might support the coupling between DNA translocation and fork unwinding.

## Highlights

• Vertical DNA movement through the MCM ring requires rotation inside the pore
• Structural asymmetries in MCM-DNA are captured during ATPase-powered translocation
• Asymmetric rotation explains selective ATPase site requirements for translocation
• The fork-stabilization complex strengthens parental-DNA engagement by the MCM

## Graphical Abstract

**Figure:**
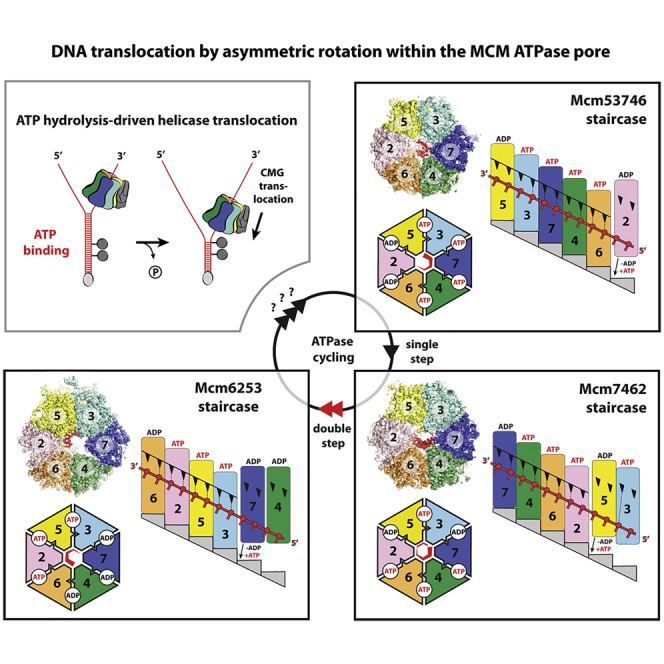

## Teaser

Eickhoff et al. used cryo-EM to image DNA unwinding by the eukaryotic replicative helicase, Cdc45-MCM-GINS. As the hexameric MCM ring hydrolyses ATP, DNA is spooled asymmetrically around the ring pore. This asymmetry explains why selected ATPase sites are essential for DNA translocation. Understanding DNA unwinding informs on replication fork progression.

## Introduction

Chromosome duplication is catalyzed by the replisome, a multi-subunit complex that combines DNA unwinding by the replicative helicase and synthesis by dedicated polymerases (Pellegrini and Costa, 2016). During eukaryotic replication, the helicase function is provided by the Cdc45-MCM-Go-Ichi-Ni-San (GINS) (CMG) assembly comprising Cdc45, GINS, and a hetero-hexameric motor known as the MCM complex (minichromosome maintenance, formed by the Mcm2-3-4-5-6-7 subunits) (Ilves et al., 2010, Moyer et al., 2006). MCM belongs to the superfamily of AAA+ ATPases (ATPases associated with various cellular activities), which contain bipartite-active sites with catalytic residues contributed by neighboring subunits (Neuwald et al., 1999). The process of CMG formation is best understood in budding yeast, mainly due to *in vitro* reconstitution studies (Deegan and Diffley, 2016). During the G1 phase of the cell cycle, MCM is loaded as an inactive double hexamer around duplex DNA (Abid Ali et al., 2017, Evrin et al., 2009, Noguchi et al., 2017, Remus et al., 2004). The switch into S phase promotes the recruitment of Cdc45 (Deegan and Diffley, 2016, Itou et al., 2015, Labib, 2010) and GINS (Deegan et al., 2016, Muramatsu et al., 2010, Zegerman and Diffley, 2007), promoting origin DNA untwisting by half a turn of the double helix (Douglas et al., 2018). Recruitment of the firing factor Mcm10 leads to replication fork establishment, which involves three concomitant events, including (1) activation of the ATP hydrolysis function of MCM, (2) unwinding of one additional turn of the double helix, and (3) ejection of the lagging strand template (Douglas et al., 2018, Lõoke et al., 2017). How CMG activation promotes eviction of the lagging strand template from the MCM pore is unclear, although it is known that extensive DNA unwinding requires replication protein A (RPA) (Douglas et al., 2018, Kose et al., 2019). The isolated CMG is a relatively slow helicase (Ilves et al., 2010), yet cellular rates of DNA replication can be achieved *in vitro* in the presence of fork-stabilization factors Csm3-Tof1 and Mrc1 (Yeeles et al., 2017). Despite these advances, a complete understanding of DNA fork unwinding and of fast and efficient replisome progression is still lacking (Abid Ali and Costa, 2016, Yeeles et al., 2017).

Mechanistic models for helicase translocation have been proposed in the past, based on streamlined systems (Lyubimov et al., 2011). For example, crystallographic and cryo-electron microscopy (EM) work on substrate-bound homo-hexameric ring-shaped helicases help explain how nucleic acid engagement can be modulated by the nucleotide state around the six nucleoside triphosphate (NTP) hydrolysis centers (Enemark and Joshua-Tor, 2006, Gao et al., 2019, Itsathitphaisarn et al., 2012, Thomsen and Berger, 2009). In most structures, five subunits form a right-handed staircase around the nucleic acid substrate and a sixth protomer (seam subunit) is found disengaged from the spiral. By morphing between six rotated copies of the same structure, a translocation mechanism can be proposed whereby sequential cycles of nucleotide binding, hydrolysis, and product release drive the successive movement of neighboring subunits (Lyubimov et al., 2011). According to the rotary model, translocation occurs with a hand-over-hand mechanism where each subunit engages, escorts, and disengages from DNA, cycling from one end of the staircase to the other (Enemark and Joshua-Tor, 2008, Lyubimov et al., 2012). Two variations of rotary cycling have been proposed, either based on a closed planar ring, where the protein staircase is formed by nucleic-acid-interacting pore loops (Enemark and Joshua-Tor, 2006, Thomsen and Berger, 2009) or based on non-planar (“lock washer”) rings with entire subunits arranged in a helical structure around DNA (Gao et al., 2019, Itsathitphaisarn et al., 2012). Homo-hexameric helicases, however, cannot be used to formally prove hand-over-hand rotary translocation, as they lack asymmetric features that would allow tracking the DNA with respect to the individual protomers within the ring (Abid Ali and Costa, 2016).

Due to its inherent asymmetry, the hetero-hexameric MCM motor seems like an ideal tool to study translocation. Gaining a molecular understanding of DNA unwinding with this system, however, has proven challenging. Promiscuous modes of DNA binding have in fact been observed for MCM, as it is capable of engaging either leading or lagging strands inside its central pore (Costa et al., 2014, Froelich et al., 2014, Georgescu et al., 2017, McGeoch et al., 2005, Rothenberg et al., 2007). In particular, it is established that yeast CMG, which translocates in a 3′ to 5′ direction (Abid Ali and Costa, 2016), unwinds DNA by threading single-stranded DNA 5′ to 3′, N to C terminal through the MCM central channel (Douglas et al., 2018, Georgescu et al., 2017). However, the inactive ADP-bound MCM double hexamer engaged to duplex DNA contains ATPase protomers that interact with both DNA strands, with Mcm7/4/6/2 binding the lagging strand (running 3′ to 5′, N-to-C), and Mcm5/3 touching the leading strand (running 5′ to 3′, N-to-C) (Abid Ali et al., 2017, Noguchi et al., 2017). When bound to a slowly hydrolysable ATP analog, *Drosophila* CMG has been observed to contact single-stranded DNA using the ATPase elements of Mcm7/4/6 (with unknown polarity) and, in a different experiment, engage a primer template junction with duplex DNA facing C-terminal MCM (opposite the polarity of replisome translocation) (Abid Ali et al., 2016, Abid Ali et al., 2017).

Attempts to image ATP-powered translocation in the active CMG thus far have only shown evidence for one mode of single-stranded DNA engagement around the MCM ring, with duplex DNA on the N-terminal side and single-stranded DNA engaged by the Mcm6/2/5/3 ATPase (Georgescu et al., 2017) (recapitulating the direction of replisome movement). However, additional rotational states in the ATP-cycling MCM complex have not been observed. One further complication to understanding nucleotide-powered DNA translocation in the CMG is that not all AAA+ sites contribute equally to unwinding. The ATP binding function of certain ATPase centers in the MCM hetero-hexamer (most prominently Mcm6) can be inactivated (by introducing a Walker A lysine to alanine, a so-called KA, change) with minimal effects on DNA unwinding *in vitro*. Conversely, the Walker A motif in the Mcm2 and Mcm5 ATPase is essential (Ilves et al., 2010). This indicates that the MCM ring is functionally asymmetric, making a strictly sequential rotary cycling (hand over hand) mechanism hard to envisage for the eukaryotic helicase. These facts, combined with the observation that the CMG has a dynamic ATPase ring gate between Mcm2 and Mcm5, have led to an alternative translocation model to rotary cycling, whereby the helicase inchworms along DNA (Abid Ali et al., 2016, Costa et al., 2011, Yuan et al., 2016). According to this inchworm, or “pump jack” model, vertical DNA movement is driven by a spiral-to-planar (extended to compressed) transition in the ATPase domain, with a gap opening and sealing at the Mcm2/5 interface to provide the power stroke for unwinding (Li and O’Donnell, 2018). However, a DNA-engaged open spiral CMG with a Mcm2-5 gap has yet to be observed, and the mechanics of ATP-hydrolysis-driven helicase progression remains to be established (Abid Ali and Costa, 2016).

To elucidate the mechanism of DNA translocation by the eukaryotic replicative helicase, we have built on DNA fork-affinity purification methods (Goswami et al., 2018) to isolate the CMG helicase undergoing extensive ATP-hydrolysis-powered fork unwinding. By using cryo-EM imaging and single-particle reconstruction, we identify four distinct ATPase states in the *Drosophila* CMG, corresponding to four modes of DNA binding. By interpolating between our structures, we can generate a model whereby vertical movement of single-stranded DNA from N- to C-terminal MCM occurs by ATPase-powered subunit movements that progress around the MCM ring. To inform this model further, we introduced single amino acid changes in the so-called arginine finger residue (Arginine-to-Alanine, or “RA”) of the six CMG catalytic centers. These alterations are known to impair ATP hydrolysis but not binding (Bochman et al., 2008). By probing the DNA helicase activity of these six CMG variants, we validate our DNA translocation mechanism. This model presents both similarities and differences with the rotary-cycling mechanism proposed for planar homo-hexameric helicases such as E1 and Rho (Enemark and Joshua-Tor, 2006, Thomsen and Berger, 2009); in particular, we find that parental duplex DNA engagement at the N-terminal front of the helicase is reconfigured in different ATPase states of the CMG. Two-dimensional EM analysis on a larger yeast replisome complex containing Mrc1-Csm3-Tof1 indicates that these factors directly associate with duplex DNA at the front of the CMG helicase. This interaction could help strengthen the coupling between single-stranded DNA translocation and the unwinding of duplex DNA at the replication fork.

## Results

### Isolation of the CMG Helicase Engaged in DNA Unwinding

**Figure 1:**
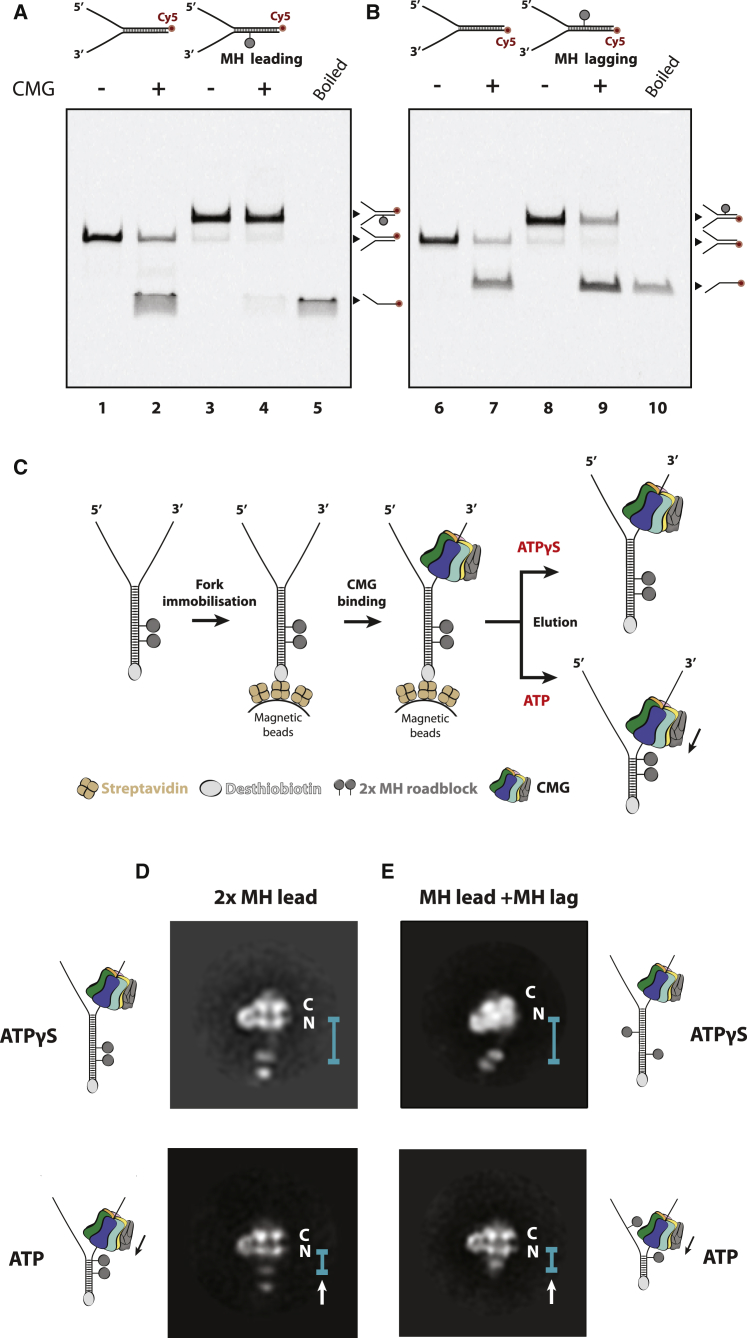
Isolation of *Drosophila melanogaster* CMG Engaged in Robust DNA Unwinding (A) Gel-based helicase assay showing that CMG cannot translocate past a DNA fork substrate containing a covalent HpaII methyltransferase (MH) roadblock on the leading strand. (B) Gel-based helicase assay showing that CMG can unwind a DNA fork substrate containing a covalent MH roadblock on the lagging strand. (C) Scheme of MH-DNA affinity purification of CMG. (D and E) 2D averages of eluted particles show that CMG maps farther from the double MH roadblock with ATPγS (D) and in closer proximity when the elution buffer contains ATP (E). A double-MH roadblock on the leading strand halts the helicase, but a single-MH roadblock on the lagging strand, upstream of a leading-strand MH roadblock, is bypassed by the advancing CMG.

To understand the molecular basis of DNA translocation by the eukaryotic replicative helicase, we focused on the structure and dynamics of CMG complexes during fork unwinding. The polarity of helicase movement (Costa et al., 2014, Douglas et al., 2018, Georgescu et al., 2017, McGeoch et al., 2005, Rothenberg et al., 2007, Trakselis et al., 2017) and the ability to bypass roadblocks on the DNA substrate (Fu et al., 2011, Kose et al., 2019, Langston and O’Donnell, 2017, Langston et al., 2017) have been the subject of debate in recent years, with suggestions that CMG helicases from different species might move in opposite directions (Trakselis et al., 2017). However, it is now established that active translocation of CMG in an *in vitro* reconstituted assay must satisfy three criteria: (1) the N-terminal side of the MCM ring should face the fork nexus (at least with yeast proteins) (Douglas et al., 2018, Georgescu et al., 2017), (2) the helicase should stall upon encountering a stable protein roadblock on the leading strand template (the translocation strand; Figure 1A), and (3) the helicase should bypass a protein roadblock on the lagging strand template (as shown in Figure 1B) (Fu et al., 2011, Kose et al., 2019).

To isolate the DNA-engaged form of the helicase, we used desthiobiotinylated DNA forks bound to streptavidin-coated magnetic beads as bait to capture purified recombinant budding yeast CMG particles. Binding was performed in the presence of ATPγS, a slowly hydrolysable ATP analog that promotes DNA engagement but not unwinding (Ilves et al., 2010, Petojevic et al., 2015). To monitor translocation and retain particles on the DNA, we introduced two HpaII methyltransferases (MH) roadblocks on the duplex DNA, covalently linked to the translocation strand, spaced 33 and 48 base pairs (bp) from the fork nexus, respectively. Using two MH adducts provides a distinguishing “double dot” feature that can serve as a fiducial for single-particle EM (Figure 1C). When ATPγS was supplemented in the biotin elution buffer, we obtained CMG averages with the N-terminal side facing the MH cross-linked duplex DNA (Figure S1), recapitulating the polarity of DNA fork engagement observed previously for yeast CMG (Douglas et al., 2018, Georgescu et al., 2017). This finding indicates that our fork-affinity purification method selects yeast CMG particles with a substrate engagement mode previously shown to be productive for helicase translocation (Douglas et al., 2018). Under these conditions, helicase assemblies are spaced from the MH by 10-15 nm and seemingly oscillate with respect to the MCM pore (Figure S1). When biotin elution was instead performed with an ATP buffer to promote translocation, 100% of yeast ATP-CMG particles with visible DNA roadblocks were found in contact with the MH (Figure S1). The CMG helicase stopped by a MH adduct on the DNA is an interesting target in and of itself for future investigation.

We reasoned that DNA engagement for a helicase stalled at a roadblock might not reflect a *bone fide* translocation mode of MCM-DNA engagement. To circumvent this problem, we switched to *Drosophila* CMG. Magnetic tweezers analysis has revealed that single CMG molecules can advance, pause, slide backward, and advance again when bound to a DNA fork (Burnham et al., 2019), increasing our chances of capturing not only stalled but also translocating forms of the CMG. By reconstructing distinct DNA binding modes and correlating them to unique ATP-occupancy states, we set out to establish the mechanism of helicase translocation (and possibly pausing) by the CMG. Similar to the experiments with yeast CMG (Georgescu et al., 2017), EM on *Drosophila* ATPγS*-*CMG revealed MH roadblocks spaced by 10–15 nm from the N-terminal MCM pore (Figures 1D, 1E, and S1). When biotin elution was instead performed with ATP buffer that promotes translocation, *Drosophila* CMG particles were found at a variety of distances, closer to (but not in contact with) the roadblock, while the mobility of the methyltransferase pointers was significantly constrained. This configuration is compatible with fork-nexus engagement and DNA unwinding by the advancing CMG helicase (Figures 1D, 1E, and S1; Video S1). The observation that both yeast and *Drosophila* CMG translocate on DNA with the N-terminal (and not the C-terminal) MCM face first is important, as it settles a long-standing controversy in the field (Trakselis et al., 2017). Because ATP-CMG showed proximity to but not direct contact with the leading-strand roadblock, we deemed *Drosophila* CMG a suitable target for high-resolution structural analysis of ATP-hydrolysis-driven translocation.

Video S1. CMG Translocation Visualized by EM Using Protein Roadblocks on DNA, Related to Figure 1 Direct EM evidence of CMG translocation using DNA with covalently linked methyltransferase roadblocks/fiducials. While CMG only loosely engages the DNA fork when incubated with ATPγS, it translocates toward the methyltransferase roadblocks when the nucleotide is switched to ATP. Duplex DNA movement, tracked by visualizing methyltransferase, is restrained when the CMG is unwinding DNA.

To confirm that the CMG helicase can engage in vigorous unwinding in our experimental conditions, we sought to visualize the bypass of a roadblock on the lagging-strand template. To this end we repeated the DNA-affinity purification and EM experiments using a fork with the first MH roadblock cross-linked to the lagging strand, 29 bp downstream of the nexus and 15 bp upstream of a leading-strand roadblock. The resulting 2D averages derived from sample eluted in ATP buffer yielded one lone methyltransferase adduct proximal to the CMG complex, as would be expected for a helicase that has translocated past the lagging-strand block, but not the downstream leading-strand adduct (Figures 1E and S1). Our data match the observation that the CMG helicase is not halted by a covalent methyltransferase roadblock on the lagging-strand template (Figures 1A and 1B) (Fu et al., 2011, Kose et al., 2019). In summary, visual analysis of our helicase-DNA complex satisfies criteria that constitute processive DNA unwinding by the CMG, including lagging-strand but not leading-strand roadblock bypass (Fu et al., 2011, Kose et al., 2019). Furthermore, we show for the first time that *Drosophila* CMG, like yeast, translocates with the N-terminal tier of MCM first (Douglas et al., 2018, Georgescu et al., 2017). Promiscuous polarity of DNA binding has implications for the mechanism of replication fork establishment, addressed in the Discussion section.

### Cryo-EM Reveals Four DNA Binding Modes around the ATPase-Ring Pore

**Figure 2:**
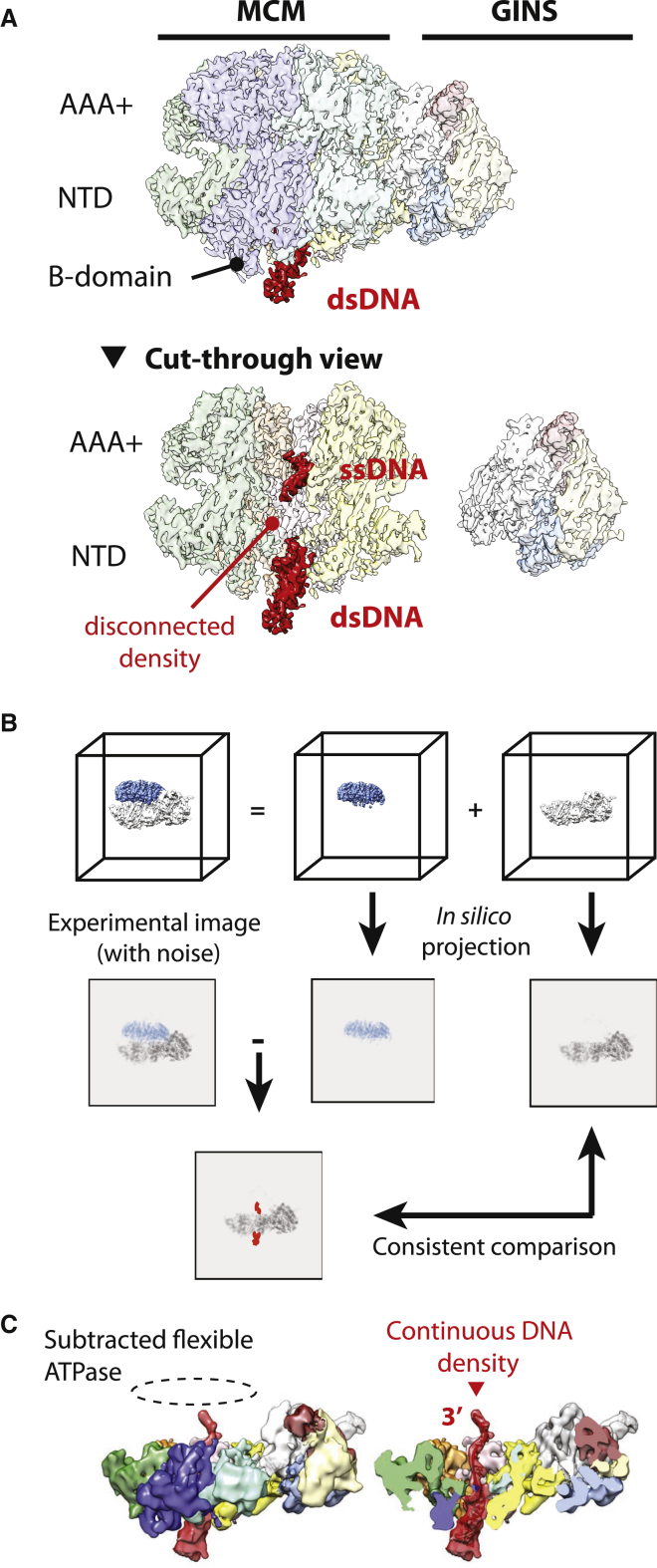
Signal Subtraction Reveals Flexibility in the AAA+ Domain of the CMG (A) Side view and cut-through side view of CMG in state 1. Duplex DNA enters the N-terminal side of the CMG and single-stranded DNA is bound by the AAA+ domain. The DNA density at the fork junction is discontinuous, indicating that CMG moves with respect to DNA. (B) Scheme for signal subtraction of the AAA+ ATPase tier of the CMG. (C) 3D class of AAA+ ATPase-subtracted CMG particles reveals a continuous density between duplex and single-stranded DNA at the fork junction. This indicates that the ATPase domain in state 1 can likely occupy different states.

To understand DNA binding by the CMG helicase during translocation, we collected cryo-EM data of the *Drosophila* CMG-DNA-MH (leading) complex in conditions that promote fork unwinding (Figure S2; Table S1). Following 3D classification and refinement approaches, we first focused our analysis on the most populated structural state (state 1, 3.46-Å resolution; Figures S2 and S3). As previously described for the yeast CMG imaged on a pre-formed fork in ATP buffer (Georgescu et al., 2017), we could clearly visualize duplex DNA entering a B-domain antechamber on the N-terminal side of the MCM ring pore (Figure 2A). Initial reconstruction efforts led to cryo-EM maps with disconnected duplex to single-stranded DNA density (Figure 2A). However, signal subtraction (Bai et al., 2015) of the ATPase domain (Figure 2B) followed by 3D classification allowed us to visualize GINS-Cdc45 and N-terminal MCM encircling duplex DNA, which now appeared connected to a single-stranded DNA density in the pore of the subtracted ATPase (Figure 2C). This exercise allows us to draw two conclusions. First, because information on DNA polarity can be extracted from mapping major and minor grooves in the double helix, we can tell that the single-stranded feature in the ATPase channel corresponds to the leading-strand template, with 3′ facing the MCM C terminus (also observed in the yeast CMG-DNA models; Georgescu et al., 2017, Goswami et al., 2018). Second, because signal subtraction of the ATPase tier improved residual particle averaging and hence the DNA map, we infer that the subtracted domain must undergo significant structural changes with respect to DNA.

**Figure 3:**
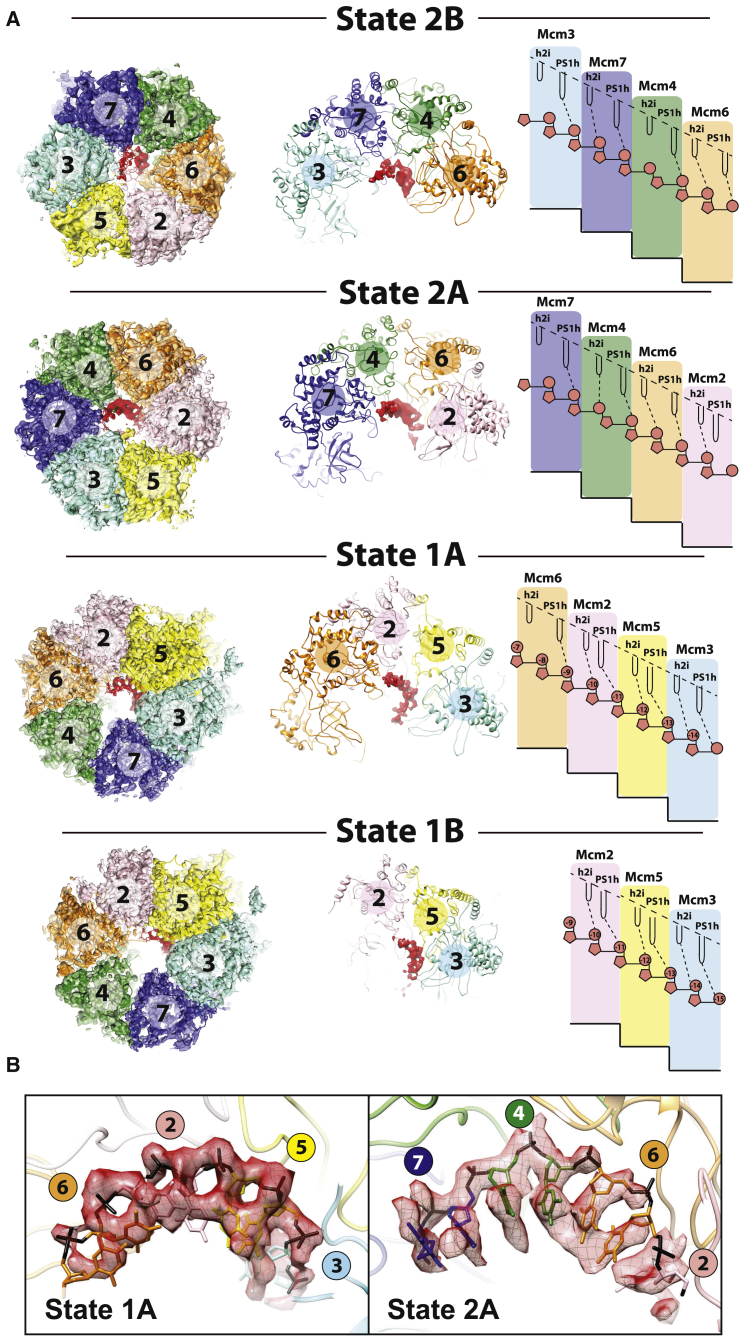
Distinct DNA Binding States around the MCM Ring in the Translocating CMG (A) Four distinct DNA binding states for the translocating CMG. In states 2B, 2A, and 1A, four ATPase protomers contain DNA-interacting pore loops arranged as a staircase. In state 1B only three protomers contact DNA forming a pore-loop spiral. Both helix 2 insertion (h2i) as well as Pre-sensor 1 beta hairpin (PS1h) pore loops contact DNA, resulting in two nucleotide contacts per protomer. (B) Detail of the cryo-EM density assigned to DNA shows that individual phosphate groups can be resolved in the single-stranded DNA region of the DNA fork, allowing us to count the number of nucleotides contacted by each ATPase protomer.

To understand how conformational transitions in the ATPase ring modulate DNA engagement inside the MCM cavity, we performed extensive three-dimensional classification on the non-subtracted particle dataset (Figure S2). These efforts led us to identify a CMG-DNA “state 2” (4.23-Å resolution; Figure S3). The MCM architectures in states 1 and 2 differ drastically, as the ATPase shifts *en bloc* with respect to the N-terminal tier, translating by 8 Å in a direction perpendicular to the MCM pore (Figure S3). To identify any additional states within the two major 3D classes, we performed further classification focused on the ATPase domains (Figures S2 and S3). This analysis led us to identify four different DNA engagement modes of the CMG, with global resolutions ranging from 3.7 to 4.5 Å. In these structures, DNA binding involves previously described ATPase pore loops named the PreSensor 1 hairpin (PS1h) and helix 2 insertion (h2i) (Abid Ali and Costa, 2016). In three of these states, ATPase pore loops from a set of four neighboring protomers form a right-handed staircase spiraling around single-stranded DNA (Figures 3 and S4). These protomers are Mcm6/2/5/3 (state 1A), Mcm2/6/4/7 (state 2A) and Mcm6/4/7/3 (state 2B), respectively. In a fourth state (state 1B) only three protomers contact single-stranded DNA (Mcm2/5/3; Figure 3A).

**Figure 4:**
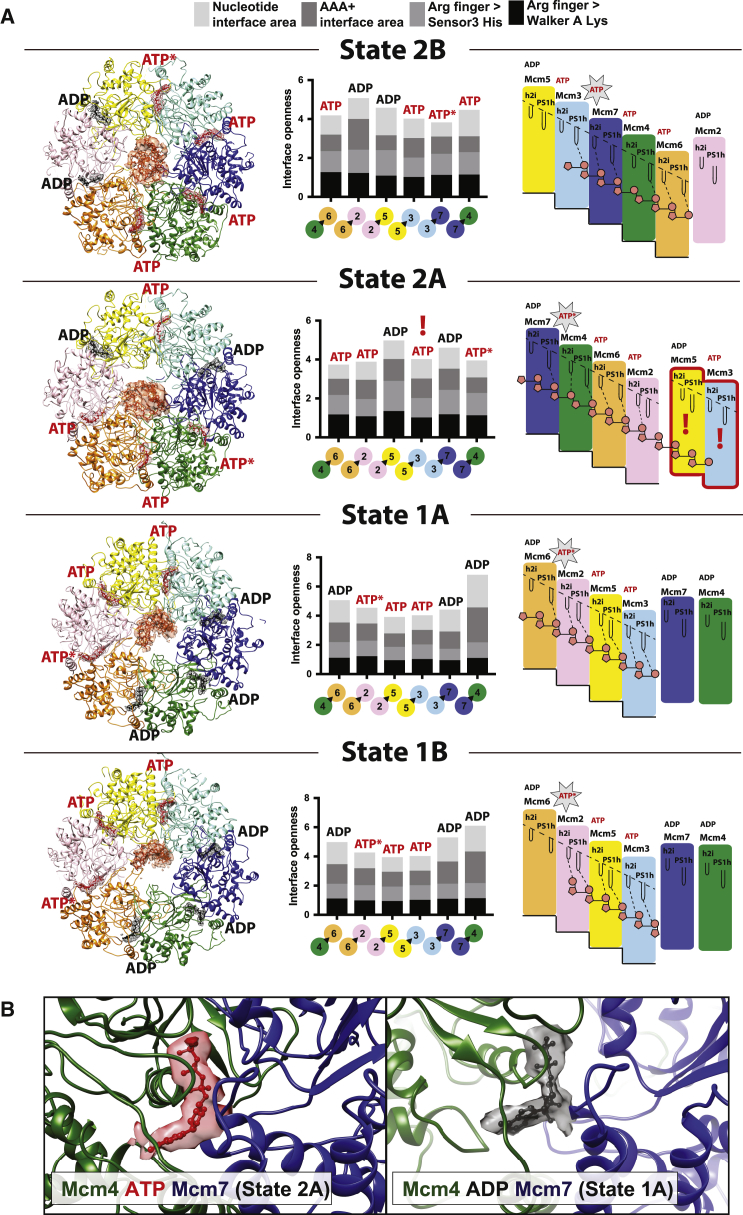
MCM Nucleotide Occupancy in Various DNA Binding States of the CMG Helicase (A) ATP at inter-protomer interfaces is shown in red. ADP is shown in black. ATP binding is observed in tighter ATPase interfaces, and ADP binding correlates with interface loosening. Inter-protomer openness is established by measuring the (1) buried surface area between nucleotide and opposed Arg Finger subunit, (2) buried surface area between neighboring ATPase domains, (3) distance between Arg finger and Sensor 3 His, and (4) distance between Arg finger and Walker A Lys. ATPase staircase formation is promoted by ATP binding. ATP hydrolysis sites are postulated to map between the two C-terminal subunits in the ATPase spiral. The C-terminal subunit in the spiral is ADP bound as are the non-engaged seam subunits. One exception (marked with a red exclamation mark) is Mcm3 (seam subunit II) in state 2A, where Mcm3 is ATP bound and tightly bound to Mcm5 (seam subunit I). This feature, we argue, contributes to the functional asymmetry within the MCM ring of the CMG helicase. Star indicates ATPase-competent site. Black triangles indicates arginine fingers. (B) A tight (ATP interacting) AAA+ interface between Mcm4 and Mcm7 (left) and a relaxed (ADP interacting) state of the same interface.

The resolution of our maps is sufficient to visualize phosphate bumps in the DNA backbones, allowing us to count contacts of two nucleotides per protomer along the single-stranded DNA stretch (Figures 3B and 3C). In state 1A, Mcm5 is found at the C-terminal end of the ATPase staircase but does not use the PS1h or h2i motifs to contact DNA. Mcm2 appears detached from the ATPase staircase in this state, poised midway between the top and bottom of the ATPase spiral (Figure 4A). Following nomenclature proposed for other hexameric ATPases, we refer to this disengaged ATPase domain as a “seam subunit” (de la Peña et al., 2018, Thomsen and Berger, 2009). State 1B appears to be virtually identical to state 1A except that the Mcm6 PS1h does not contact DNA. This configuration might reflect a corrupted state in which the DNA became disengaged during sample handling or freezing, or could represent a physiologically relevant form of the helicase (perhaps a stalled state). In states 2A and 2B, two neighboring AAA+ modules (which we refer to as seam subunits I and II) appear detached from the staircase. With our results, we provide the first direct evidence that, under conditions that promote DNA translocation, the leading strand template can touch different ATPase protomers around the hetero-hexameric MCM ring.

### ATPase State around the AAA+ Ring

Our observations suggest a model whereby pore-loop staircase formation and DNA engagement correlate with nucleotide state in the six ATPase centers, similar to the mechanism proposed from structural studies on homo-hexameric helicases (Enemark and Joshua-Tor, 2006, Thomsen and Berger, 2009). To corroborate this model, we inspected the six ATPase sites at inter-protomer interfaces in our four states, seeking to identify ATP- and ADP-bound centers. In assigning the ATPase state, we considered three properties, including (1) the openness of the ATPase interface, (2) the active-site geometry, and (3) the cryo-EM map in the ATPase pocket. The openness of the ATPase interface was assessed by measuring the solvent-excluded surface area between protomers, or between nucleotide and the protomer that provides an arginine finger (Arg Finger) (de la Peña et al., 2018). Since the resolution in the four structures was not always sufficient to assign rotamers, active-site geometry was assessed by measuring the distance between the β carbon of the Walker-A lysine and either the sensor 3 histidine β carbon, or the Arg Finger β carbon (Figures 4A and 4B). A complete list of these measurements is reported in Table S2.

**Figure 5:**
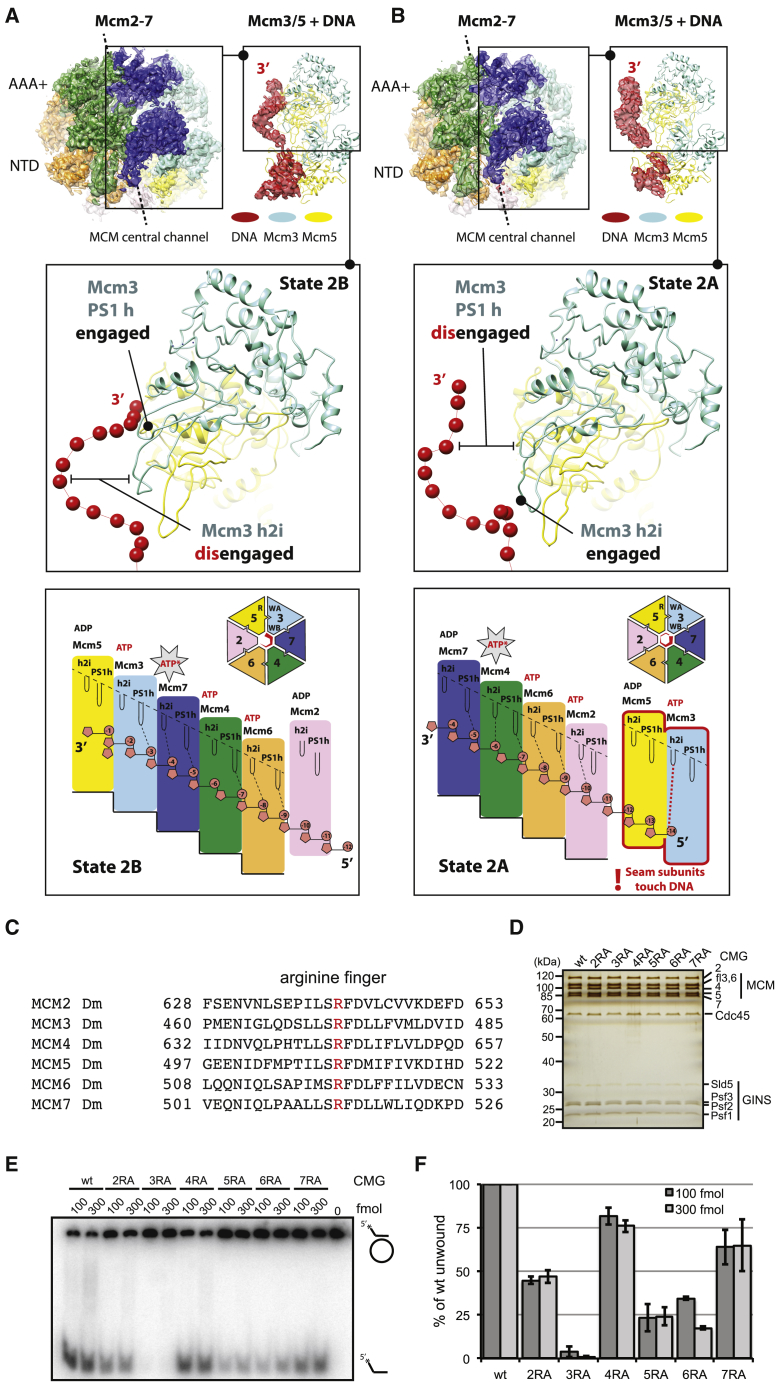
Asymmetry of DNA Binding in Two Subsequent CMG Rotational States (A) In state 2B, Mcm3 touches single-stranded DNA at the 3′ end via the PS1h pore loop, while h2i is disengaged. (B) In state 2A, Mcm3 (seam subunit II) is not part of the staircase, but is instead tightly bound to Mcm5 (seam subunit I) and engages incoming 5′ DNA on the MCM N-terminal side. This unexpected DNA interaction by seam subunit II (highlighted with a red exclamation mark), we argue, contributes to the functional asymmetry within the MCM ring of the CMG helicase. Star marks ATPase-competent site. (C) Sequence alignment of the six *Drosophila* MCM subunits. Arginine finger is highlighted in red. (D) Silver-stained gel for the six CMG variants containing single RA amino acid changes targeting the arginine finger. (E) Autoradiographs of the M13-DNA-based unwinding reaction products separated on PAGE. Indicated amounts of CMG in femtomoles were added to circular DNA. Position of double-stranded substrate and displaced oligo are indicated with DNA schematics. (F) Quantified results for four independent helicase assays. The graphs show percentage of displaced oligonucleotide as a function of protein added to reaction. Error bars indicate the standard deviations of two independent series.

Our analysis highlights a correlation between tight inter-protomer interaction, increased proximity between ATPase site elements, and ATP occupancy. Protomers that engage DNA are generally ATP bound at the N-terminal, 5′-interacting end of the AAA+ spiral and between central ATPase domains. In contrast, protomers at the C-terminal 3′-interacting end of the AAA+ spiral are ADP bound. This pattern is compatible with the previously proposed rotary, hand-over-hand model (Enemark and Joshua-Tor, 2006, Thomsen and Berger, 2009), suggesting that ATP hydrolysis occurs within the last competent ATPase site at the C-terminal end of the AAA+ staircase (Figure 4A). In three of our four structures, ATP-bound protomers are all tightly interacting and staircase engaged, and flanked by two (state 2B) or three (states 1A and 1B) ADP-bound protomers. One exception is represented by state 2A, which contains two seam subunits (Mcm5 and Mcm3) engaged in a tight AAA+ interaction around an ATP molecule (Figure 4A). Unexpectedly, ATP-Mcm3 (seam subunit II) contacts DNA through the h2i pore loop, which projects toward the incoming DNA and the N-terminal side of the MCM ring. We note that the DNA in states 2B and 2A has a register shift of one subunit, resulting in vertical DNA repositioning. In ADP-Mcm3 of state 2B, PS1h touches the 3′ end of DNA at the C-terminal end of the staircase while ATP-Mcm3 (seam subunit II of state 2A) touches the 5′ end of DNA (Figures 5A and 5B). Conversely, no direct DNA interaction can be detected for ADP-bound seam subunits in states 1A, 1B, and 2B. Thus, MCM-DNA interactions occur asymmetrically around the ring.

### Asymmetric ATP Hydrolysis Function around the MCM Ring of the CMG

In contrast to previously proposed hand-over-hand translocation models (Enemark and Joshua-Tor, 2008, Gao et al., 2019, Itsathitphaisarn et al., 2012), DNA binding around the MCM ring appears asymmetric, which could explain the asymmetry in ATPase site requirements for different hexamer interfaces (Ilves et al., 2010). Previous biochemical work on *Drosophila* CMG established that several Walker-A elements in the MCM hexamer tolerate a KA-inactivating amino acid change that is known to impair ATP binding (Ilves et al., 2010). To establish whether the functional asymmetry extends to the ATP hydrolysis function, we generated six variants of the *Drosophila* CMG complex. These variants contain an RA substitution in the Arg Finger, which is known to impair ATP hydrolysis but not binding (Figures 5C and 5D) (Bochman et al., 2008, Ilves et al., 2010). Helicase assays using these mutated CMG complexes indicate that ATP hydrolysis at the Mcm3-7 ATPase site is essential for unwinding. Conversely, impairing ATP hydrolysis at the Mcm4-6 and Mcm7-4 sites has only a minor effect on unwinding. Finally, Arg Finger substitutions at the Mcm6-2, -2-5, and -5-3 sites only have intermediate effects (Figures 5E and 5F). Notably, not all sites that strictly require ATP binding also require hydrolysis. In the Discussion section we elaborate on the correlation between structural and functional asymmetry in MCM ATPase, which together explain key features of the DNA unwinding mechanism.

### ATPase-Modulated Fork-Nexus Engagement

The structures determined here not only show a correlation between ATP binding and single-stranded DNA engagement, but also changes in the fork-nexus interaction with N-terminal MCM depending on ATPase state. In fact, in states 1A and 1B, prominent duplex DNA density is visible, primarily interacting with the B domains of Mcm5, -6, and -4 (Figure S5). Conversely, in state 2B, duplex DNA can only be seen to interact with stabilizing helicase elements such as the B domain of Mcm5, whereas in state 2A, the incoming duplex DNA is not well resolved. Two-dimensional averages of side views confirm that states 2A and 2B indeed show dynamic duplex-DNA engagement, explaining the weak density protruding from N-terminal MCM in the averaged cryo-EM volume (Figure S5). The observation that duplex DNA faces N-terminal MCM in states 2A and 2B is important because it rules out the possibility that single-stranded DNA interaction as observed in state 2 might result from binding to DNA with inverted polarity, as previously suggested (Abid Ali et al., 2016, Costa et al., 2014). Likewise, although the single-stranded/duplex DNA junction is not resolved in the states 2A and 2B, it is clearly visible in states 1A and 1B.

Interestingly, cryoSPARC (Punjani et al., 2017) refinement of a dataset with state 1-like DNA engagement (Figure S5) reveals clear density for the lagging strand DNA, departing from the DNA junction split by N-terminal beta hairpin of Mcm7 and threading through an opening between the Mcm5/3 B domains. Thus, DNA can make a ∼90° kink that positions the excluded strand near the front of the helicase (Figure S5). This observation is in striking agreement with a speculative model of the replication fork structure proposed by O’Donnell and Li, based on the analysis of the B domain architecture in the MCM ring (Georgescu et al., 2017, Li and O’Donnell, 2018). The lagging-strand density feature is notable as it has not been seen in previous *Drosophila* (Abid Ali et al., 2016) or yeast CMG assemblies (Georgescu et al., 2017, Goswami et al., 2018). We also note that the lagging-strand template is positioned in a region of the replisome complex that is known to be occupied by Pol alpha, possibly facilitating Okazaki fragment priming and parental histone redepositioning (Evrin et al., 2018, Gan et al., 2018, Sun et al., 2015). Finally, the narrow passage between Mcm3/5 B domains provides an escape route for the lagging-strand template from an antechamber of the MCM central channel (Figure S5). This route provides an explanation for how the advancing CMG helicase could bypass a protein roadblock on the lagging strand (Figure 1A) (Kose et al., 2019).

### A Duplex-DNA Binding Role for the Fork-Stabilization Factors in the Replisome

Several factors have been implicated in modulating replisome progression in difficult-to-replicate regions. For example, Mcm10, a firing factor essential for replication fork establishment, has been proposed to change the mode of CMG-fork-nexus engagement, hence facilitating lagging-strand roadblock bypass (Langston et al., 2017). However, the Mcm10-CMG interaction is dynamic and could not be characterized in previous EM imaging attempts (Douglas et al., 2018, Mayle et al., 2019). A second factor implicated in modulating replisome progression is the fork-stabilization complex, which is formed by Mrc1, Csm3, and Tof1 (the MTC) and competes for the same binding site as Mcm10 on the CMG (Douglas and Diffley, 2016). According to *in vitro* reconstitution studies with purified yeast proteins, cellular rates of DNA replication can be achieved when the reconstituted replisome is supplemented with the MTC assembly (Lewis et al., 2017, Yeeles et al., 2017). We postulated that at least some of these factors might interact with DNA at the replication fork and, perhaps, select for a substrate engagement mode productive for unwinding.

**Figure 6:**
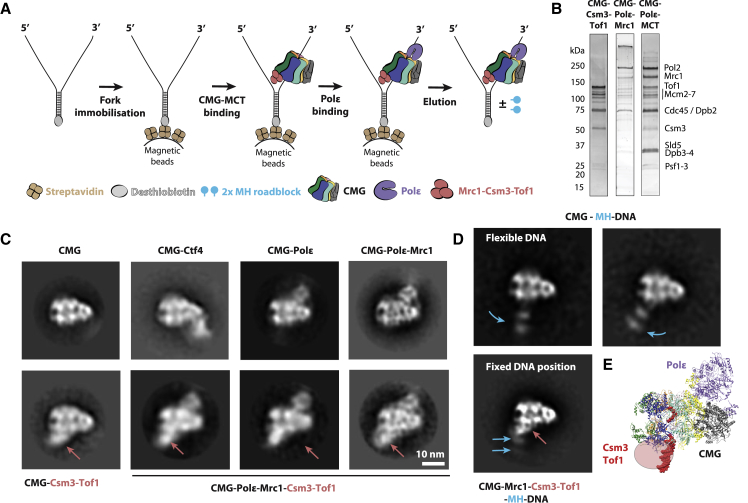
Reconstitution of a CMG-Pol Epsilon-Mrc1-Csm3-Tof1 Complex by DNA Fork-Affinity Purification (A) Purification scheme for the CMG-Pol epsilon-Mrc1-Csm3-Tof1 complex. (B) Silver-stained gels of a complex lacking Mrc1 or Csm3-Tof1, or of the full CMG-Pol epsilon-Mrc1-Csm3-Tof1 complex. (C) Two-dimensional averages of DNA-CMG-Pol epsilon-Mrc1-Csm3-Tof1 complex highlight an additional density bound to the N-terminal side of MCM (indicated by a red arrow), which is absent in control CMG or CMG-Pol epsilon preparations. The additional density is mainly provided by Csm3-Tof1, given that the N-terminal density persists in a CMG-Csm3-Tof1 complex but disappears in a CMG-Mrc1 assembly. Overlay between the full complex and the atomic structure of DNA-CMG-Pol epsilon indicates that the distinctive density feature maps in close proximity to parental DNA. (D) 2D average of ATPγS-CMG and ATPγS-CMG-Csm3-Tof1-Mrc1 bound to a fork containing two covalently linked MH roadblocks on the parental DNA. While in the absence of Csm3-Tof1-Mrc1 DNA is flexibly engaged by the helicase (as judged by the position of MH fiducials), in the presence of Csm3-Tof1-Mrc1, DNA appears fixed in one orientation. (E) Based on our data, we propose a model whereby Csm3-Tof1 touch the parental duplex DNA at the replication fork, potentially increasing coupling efficiency between single-stranded DNA translocation and fork unwinding.

To test our hypothesis, we used yeast proteins to reconstitute a Pol epsilon-CMG-Csm3-Tof1-Mrc1 replisome complex on DNA (Figures 6A and 6B), using the fork-affinity purification strategy described above. A full Pol epsilon-CMG-Csm3-Tof1-Mrc1 complex could be reconstituted on DNA-bound beads and the fork-bound complex could be eluted with biotin, in quantities sufficient for negative-stain EM analysis (Figure S6). 2D averaging revealed a mixture of different complexes, including CMG, CMG-Pol epsilon, and a class containing a distinctive protein feature on the N-terminal side of the MCM ring (Figures 6C and S6). This density maps to a position distinct from that occupied by Ctf4 (another N-terminal MCM-interacting factor; Figures 6C and S6) and disappears in a Csm3-Tof1 dropout Pol epsilon-CMG-Mrc1-DNA complex (Figures 6C and S6). Our results are consistent with the notion that Csm3-Tof1 is positioned at the front of the helicase. This model is further supported by the observation that a CMG-Csm3-Tof1 assembly retains the N-terminal feature in the absence of Mrc1 and Pol epsilon (Figures 6C and S6).

After carefully inspecting the Csm3-Tof1 density in the Pol epsilon-CMG-MTC averages, we noted that these components depart from the N-terminal tier of MCM following the same angle seen for the parental duplex DNA in our previously reported yeast Pol epsilon-CMG-DNA complex (Goswami et al., 2018) (Figures 6C–6E). This observation prompted us to postulate that the MTC complex might interact with incoming parental DNA. In line with this notion, we observed that the MTC complex could be purified using DNA-affinity methods either with a fork or blunt duplex DNA as bait (Figure S7). Dropout experiments indicate that the DNA interaction is mainly supported by Csm3-Tof1, as isolated Mrc1 could not form a stable DNA complex in our bead-based assay. This finding is further confirmed by electrophoretic mobility shift assays indicating that, compared to Mrc1, Csm3-Tof1 has higher affinity for forked or blunt duplex DNA (although the full MTC complex provides the most pronounced gel shift; Figure S7). Gel-shift assays in the presence of an anti-calmodulin binding protein (CBP) antibody (which targets Csm3-Tof1) or an anti-FLAG antibody (which is directed against Mrc1) indicate that the DNA binding function is provided by MTC factors and not by uncharacterized contaminants (Figure S7). Currently, a DNA binding function has been previously described for Mrc1 and Csm3/Tof1 homologs in *S. pombe* and humans (Sar et al., 2004, Tanaka et al., 2010, Witosch et al., 2014, Zhao and Russell, 2004). Combined with the information that Csm3-Tof1 forms a stable assembly with DNA in our fork-affinity purification assay, we suggest that these fork-stabilization factors are likely to contact parental duplex DNA in front of the helicase. In agreement with this hypothesis, although ATPγS-CMG only loosely engages parental DNA (resulting in the oscillation MH fiducials with respect to the helicase), Csm3-Tof1 significantly restrains duplex-DNA oscillation at the replication fork (Figure 6D; Video S2). Future efforts will be focused on visualizing replication-fork advancement using a replisome progression complex containing the fork-stabilization factors, Csm3-Tof1-Mrc1. These endeavors will hopefully explain whether fast and efficient replisome progression is achieved thanks to MTC contacting incoming parental DNA at the fork or alternatively because of a structural change induced in the CMG complex.

Video S2. Mrc1-Csm3-Tof1 Restrains Parental Duplex DNA Flexibility on a CMG-Bound Fork, Related to Figure 6 While DNA bound by isolated ATPγS-CMG is highly flexible, addition of Mrc1-Csm3-Tof1 restrains the parental duplex DNA in one configuration with respect to the helicase central pore. This property of the fork-stabilization complex might play a key role in increasing coupling efficiency between single-stranded DNA translocation and fork unwinding.

## Discussion

### Mechanism of ATPase-Powered DNA Translocation

**Figure 7:**
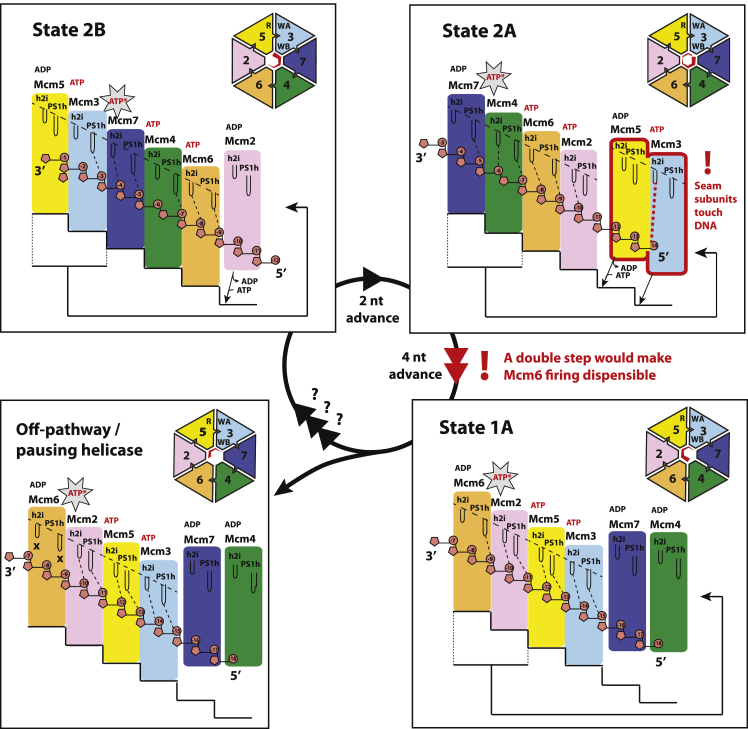
An Asymmetric Hand-over-Hand Rotational Mechanism for CMG Translocation Transition from states 2B to 2A involves release of 3′ DNA by Mcm3 PS1h and ATP binding by Mcm2, which binds to the N-terminal end of the AAA+ staircase. At the same time, Mcm3 h2i pore loop touches the incoming 5′ DNA on the N-terminal side of MCM. In state 2A, seam subunits Mcm5 and Mcm3 sandwich ATP. ATP-Mcm5 association with the N-terminal side of the AAA+ staircase would drag ATP-Mcm3 along, causing detachment of two subunits from the C-terminal end of the spiral. As a result, Mcm6 is found at the C-terminal end of the AAA+ staircase (state 1A). As Mcm5-3 binding to the ATPase spiral may occur en bloc, this would render ATP hydrolysis by Mcm6 dispensable. We speculate that state 1B, where Mcm6 is disengaged from DNA, might be off pathway and represent a pausing state. Alternatively, it might represent a corrupted version of state 1A, which partially disassembled from DNA during handling or freezing. Star marks ATPase-competent site.

In the present study, we have analyzed the CMG helicase on a model DNA fork using conditions that allow for ATP-dependent DNA translocation (Ilves et al., 2010). As observed in other hetero-hexameric AAA+ motors (de la Peña et al., 2018, Dong et al., 2019), and inferred from studies on homo-hexameric helicases (Enemark and Joshua-Tor, 2006, Enemark and Joshua-Tor, 2008, Lyubimov et al., 2011, Thomsen and Berger, 2009), the translocation substrate can be found in different positions around the MCM ring as the helicase unwinds the fork. In three of our four structures (states 1A, 2A, and 2B), single-stranded DNA is engaged by four ATPase subunits via a set of pore loops arranged in a right-handed spiral (Figure 3A). In general, ATP binding appears to promote DNA binding and establish the AAA+ staircase structure, while DNA-free subunits are instead ADP bound. One might argue that, from our structural data alone, we cannot rule out that ATPase firing is stochastic (Abid Ali and Costa, 2016). However, rotary cycling appears to make physical sense, resulting in efficient vertical movement from N- to C-terminal MCM, with the 3′ end of DNA leading the way inside the ring channel (as exemplified in the states 2B-to-2A transition in Figures 5 and 7). Importantly, our mutational data provide strong evidence in support of this translocation mechanism. In our model, translocation involves a clockwise rotation, with the 3′ DNA moving toward the observer when the motor is viewed from the C-terminal end.

To represent this sequential rotary cycling movement, a video can be generated (Video S3) by morphing between states 2B→2A→1A. According to the “canonical” rotary hand-over-hand mechanism, an ATP-bound subunit would engage the substrate at the N-terminal end of the staircase, hydrolyse ATP at the penultimate position of the staircase, remain ADP bound at the top of the staircase, and exchange the nucleotide as it transitions from the top to the bottom of the staircase (seam subunits). This scheme is followed upon transitioning from states 2B to 2A, where we can model a one-subunit step with a two-nucleotide advancement (Figures 5A, 5B, and 7). In this transition, Mcm3 disengages from the C-terminal end of the spiral as a consequence of ATP hydrolysis, while ATP-Mcm2 joins the DNA-interacting AAA+ staircase from the N-terminal end. Consistent with this modeled transition, we show that ATP hydrolysis at the Mcm3-7 interface is essential for translocation (Figure 5), given that an Mcm3 Arg Finger RA change abrogates unwinding. Conversely, ATP binding (but not hydrolysis) at the Mcm5-3 interface is strictly required for activity, given that a Walker-A KA mutation in Mcm3 abrogates translocation (Ilves et al., 2010), whereas a Mcm5 RA change only partially affects DNA unwinding. Collectively, this evidence supports a model in which Mcm53 can transition as an ATP-stabilized rigid dimer, as predicted by morphing between states 2B and 2A (Figure 7).

Video S3. Mechanism of ATPase-Driven DNA Translocation by the CMG Helicase, Related to Figure 7 Mechanism of ATP-hydrolysis driven single-stranded-DNA translocation by the CMG helicase. Asymmetric DNA engagement around the MCM ring explains the asymmetric ATPase requirements for DNA unwinding.

Modeling of the subsequent state 2A-to-1A transition reveals a two-subunit (four nucleotide) step, promoted by ATP binding to Mcm5, which tightens the Mcm25 interface and causes the Mcm5/3 dimer to join the AAA+ staircase (Figure 7). This morphed transition is supported by the observation that an Mcm5 Walker-A KA change abrogates unwinding. Conversely, ATP hydrolysis at the C-terminal end of the staircase appears dispensable, given that Mcm7 tolerates an Arg Finger RA mutation with minimal effect on unwinding (Ilves et al., 2010). By combining our DNA unwinding and structural data, we establish that ATP hydrolysis within the MCM ring is likely to occur at the third inter-subunit interface, counting from the 5′-interacting, N-terminal end of the AAA+ staircase. As our structural and DNA unwinding data support a two-subunit (i.e., four-nucleotide) step upon transition from states 2A to 1A, we note that the Mcm6/4 interface would never be found in the ATP-hydrolysis-competent third position of the AAA+ staircase. This observation leads to the prediction that ATPase function might be dispensable at this interface, and indeed, an Mcm4 Arg Finger RA change manifests near-wild-type DNA unwinding activity in our assay, mirroring the effect of an Mcm6 KA change targeting the same inter-protomer interface.

At present, we only observe three rotational states around the MCM ring. We readily concede that other translocation intermediates might exist. Crucially, however, we note that with the modeled transitions between our observed rotational states we can explain why selected ATP hydrolysis (Mcm3 Arg Finger) and ATP binding (Mcm3 and Mcm5 Walker-A; Ilves et al., 2010) functions are strictly required to support DNA translocation. At the same time, our model provides a rationale for why ATP binding (Mcm6 Walker A) (Ilves et al., 2010) or hydrolysis (Mcm4 Arg finger) is dispensable in other sites. Overall, the modeled transitions are supported by available mutational data and collectively describe not only a mechanism for the ATP-powered translocation of single-stranded DNA by the CMG, but also explain the functional asymmetry of the MCM ring. In addition, the unique protein-DNA contacts in state 2A explain how a two-subunit (four nucleotide) step can physically occur. Unlike states 1A and 2B, where seam subunits are disengaged from any DNA interaction, in state 2A, the h2i pore loop of Mcm3 engages DNA upstream of the staircase-engaged DNA (Figure 5B). Upon transitioning from state 2B to state 2A, Mcm3 lets go of the PS1h-mediated staircase-DNA interaction, while the h2i pore loop reaches for single-stranded DNA entering the N-terminal side of the MCM ring. The subsequent exchange of ADP for ATP by Mcm5 causes the double step by promoting the *en bloc* engagement (at the N-terminal end) of Mcm5/3 with the four-subunit DNA staircase. In this state both Mcm5 and Mcm3 subunits fully engage four nucleotides of single-stranded DNA (Figure 7). These actions generate rotational movement around the motor of the CMG and also vertical movement of single-stranded DNA through the MCM ring. While we do see a small gap at the Mcm2-5 interface arising in state 2A, the ring remains planar and compressed, indicating that a spiral-to-planar transition is not required for helicase advancement, as has instead previously been suggested (Abid Ali et al., 2016, Yuan et al., 2016)

Strikingly, fork-nexus engagement is altered in different rotational states, as observed on the N-terminal side of the MCM ring (Figure S5). We show that the fork-stabilization factors Csm3-Tof1 bind to duplex DNA *in vitro*, associate with the N-terminal domain of MCM, and align with incoming parental DNA at the fork (Figures 6C–6E, S6, and S7). We postulate that these factors might play a role in selecting and stabilizing productive substrate engagement for CMG translocation, hence strengthening the coupling between DNA rotation and unwinding at the fork. Coherent with this notion, Tof1 and Csm3 have been implicated in inhibiting excessive fork rotation and precatenation, hence preventing genomic instability (Schalbetter et al., 2015).

### Implications for Duplex-DNA Melting

Our structural data have significant implications for the mechanism of ATP-dependent origin DNA melting at the onset of replication. The structures presented here provide conclusive evidence that the same AAA+ elements in the MCM ring can touch DNA in opposing orientations between the inactive (MCM double hexamer) and active (CMG) helicase forms. In the MCM double hexamer, the ATPase pore loops of Mcm3 and Mcm5 touch the leading-strand template (running 3′-to-5′ from the C- to N-terminal side), whereas Mcm2/4/6/7 ATPase pore loops contact the lagging-strand template (running 5′-to-3′ from the C- to N-terminal side of the MCM pore) (Abid Ali et al., 2017, Noguchi et al., 2017). Conversely, in ATP-CMG state 2A, the same pore loops from the six MCM subunits solely touch the leading-strand template, running 3′-to-5′ from C to N (Figure S5).

To understand the relevance of this finding, it is useful to describe the steps that lead to origin activation, as is currently understood. The duplex-DNA-loaded MCM forms a double hexamer and is inactive; however, it undergoes a conformational change upon recruitment of the GINS and Cdc45 activators, which promotes ADP release and ATP binding, and the concomitant untwisting of duplex DNA by half a turn of the double helix. A transient interaction with Mcm10 subsequently leads to the activation of the ATP hydrolysis function and ejection of the lagging strand template from the MCM ring pore. Concomitant with these two events, the CMG unwinds one whole turn of the double helix (Douglas et al., 2018) (Figure S5).

Our data provide a rationale for how the lagging strand can be disengaged and actively expelled from the ATPase ring pore, as ATP-dependent, leading-strand translocation ensues. One complete round of ATPase firing around the MCM ring would unwind one turn of the double helix and cause the leading-strand template to engage, in different steps, all AAA+ protomers around the ring. This event would occur irrespective of whether the ATP hydrolysis is stochastic or sequential, causing the translocation strand to “sweep” across the inner perimeter of the MCM pore, dislodging the lagging strand for its unique ATPase binding site (the Mcm4/7/3 h2i interaction). Mobilization of the leading strand inside the MCM ring would in turn put the lagging strand template into a high-energy state favoring displacement. At this stage, lagging-strand escape from the helicase pore would be allowed by the opening of an MCM ring gate, which has been proposed to be Mcm10 mediated (Figure S5) (Douglas et al., 2018, Douglas and Diffley, 2016, Lõoke et al., 2017). An MCM single-stranded DNA binding element (the MSSB) could play an important role in this process. Mapping to a position underneath the h2i pore loops on the N-terminal domain of the adjacent Mcm4/6/7 protomers, the MSSB could stabilize the lagging-strand template as it is being displaced from its AAA+ binding site, *en route* to ejection from the helicase ring pore (Froelich et al., 2014). Future structural characterization of the origin unwinding reaction will be key for a full mechanistic understanding of this process.

While DNA unwinding by the CMG occurs with 3′-to-5′ polarity, multiple lines of evidence indicate that the MCM motor is capable of interacting with DNA in either direction (Costa et al., 2014, Froelich et al., 2014, Georgescu et al., 2017, McGeoch et al., 2005, Rothenberg et al., 2007). We note that promiscuity of substrate binding by ATPase pore loops is not a novelty for AAA+ motors. One example is the Rpt1-6 motor of the proteasome, which can translocate with equal efficiency on polypeptides threaded into the pore with N-to-C or C-to-N polarity. According to our CMG model, the promiscuity of DNA engagement is a core feature in the mechanism of ATP-dependent origin DNA unwinding. This model reconciles 15 years of diverging findings on the directionality of DNA engagement by the replisome (Froelich et al., 2014, Georgescu et al., 2017, Goswami et al., 2018, McGeoch et al., 2005, Rothenberg et al., 2007, Trakselis et al., 2017).

## STAR★Methods

### Key Resources Table

**Table:** 

REAGENT or RESOURCE	SOURCE	IDENTIFIER
**Antibodies**		

Anti-CBP antibody	Sigma-Aldrich	RRID: AB_10743822
Anti-FLAG antibody	Sigma-Aldrich	RRID: AB_2811010

**Chemicals, Peptides, and Recombinant Proteins**		

3X FLAG peptide	Sigma	F4799
Anti-FLAG M2 affinity gel	Sigma	A2220
Calmodulin-Sepharose 4B	GE Healthcare	17-0529-01
cOmplete, EDTA-free	Roche	5056489001
yeast CMG	Zhou et al., 2017	N/A
*Drosophila* CMG	Ilves et al., 2010	N/A
Mrc1	Yeeles et al., 2017	N/A
Csm3/Tof1	Yeeles et al., 2017	N/A
Ctf4	Gambus et al., 2009	N/A
Pol epsilon(exo-)	Goswami et al., 2018	N/A
M.HpaII	NEB	M0214S

**Deposited Data**		

*Drosophila* CMG-DNA state 1A cryo-EM map	This paper	EMD-4785
*Drosophila* CMG-DNA state 1A PDB coordinates	This paper	6RAW
*Drosophila* CMG-DNA state 1B cryo-EM map	This paper	EMD-4786
*Drosophila* CMG-DNA state 1B PDB coordinates	This paper	6RAX
*Drosophila* CMG-DNA state 2A cryo-EM map	This paper	EMD-4787
*Drosophila* CMG-DNA state 2A PDB coordinates	This paper	6RAY
*Drosophila* CMG-DNA state 2B cryo-EM map	This paper	EMD-4788
*Drosophila* CMG-DNA state 2B PDB coordinates	This paper	6RAZ

**Experimental Models: Cell Lines**		

High Five cells	Thermofisher	B855-0202
Sf9 cells	Thermofisher	A38841

**Experimental Models: Organisms/Strains**		

yJCZ3 (yeast CMG purification)	Zhou et al., 2017	N/A
yAE99 (Pol epsilon exo-)	Goswami et al., 2018	N/A
yAE48 (Csm3/Tof1 purification)	Yeeles et al., 2017	N/A
yJY32 (Mrc1 purification)	Yeeles et al., 2017	N/A

**Oligonucleotides**		

A list of oligonucleotides is provided in Table S3.		N/A

**Recombinant DNA**		

pFastBac1 Mcm2	Ilves et al., 2010	N/A
pFastBac1 Mcm3	Ilves et al., 2010	N/A
pFastBac1 Mcm4	Ilves et al., 2010	N/A
pFastBac1 Mcm5	Ilves et al., 2010	N/A
pFastBac1 Mcm6	Ilves et al., 2010	N/A
pFastBac1 Mcm7	Ilves et al., 2010	N/A
pFastBac1 Cdc45	Ilves et al., 2010	N/A
pFastBac1 Psf1	Ilves et al., 2010	N/A
pFastBac1 Psf2	Ilves et al., 2010	N/A
pFastBac1 Psf3	Ilves et al., 2010	N/A
pFastBac1 Sld5	Ilves et al., 2010	N/A
pFastBac1 RA Mcm2	This study; Ilves et al., 2010	N/A
pFastBac1 RA Mcm3	This study; Ilves et al., 2010	N/A
pFastBac1 RA Mcm4	This study; Ilves et al., 2010	N/A
pFastBac1 RA Mcm5	This study; Ilves et al., 2010	N/A
pFastBac1 RA Mcm6	This study; Ilves et al., 2010	N/A
pFastBac1 RA Mcm7	This study; Ilves et al., 2010	N/A

**Software and Algorithms**		

RELION v2.1 and v3	Scheres, 2012	https://www2.mrc-lmb.cam.ac.uk/relion/index.php?title=Main_Page
MotionCor2	Zheng et al., 2017	https://msg.ucsf.edu/em/software/motioncor2.html
cryoSPARC v2	Punjani et al., 2017	https://www.nature.com/articles/nmeth.4169
Coot v0.8.8	Emsley et al., 2010	http://scripts.iucr.org/cgi-bin/paper?S0907444910007493
PHENIX v1.13	Adams et al., 2010	http://www.phenix-online.org/
UCSF Chimera	UCSF Resource for Biocomputing, Visualization, and Informatics	https://www.cgl.ucsf.edu/chimera/

### Lead Contact and Materials Availability

Further information and requests for resources and reagents should be directed to and will be fulfilled by the Lead Contact, Alessandro Costa (alessandro.costa@crick.ac.uk). Material will be made available upon reasonable request. This study did not generate new unique reagents.

### Experimental Model and Subject Details

#### Yeast Expression

Yeast proteins were purified from *Saccharomyces* cerevisiae strains (genotypes: MATa ade2-1 ura3-1 his3-11,15 trp1-1 leu2-3,112 can1- 100 bar1::Hyg pep4::KanMX or MATa pep4::KanMx4 bar1::Hph-NT1 yJCZ1: MATα pep4::KanMx4 bar1::Hph-NT1 ade2-1::pJCZ3 (ADE2)) containing integrated expression constructs and grown at 30°C in YEP media supplemented with 2% raffinose.

#### Baculovirus Expression

*Drosophila melanogaster* proteins were purified from baculovirus-infected female High-five cells incubated at 27°C, as previously described (Abid Ali et al., 2016, Ilves et al., 2010).

### Method Details

#### Cloning and Construction of Baculoviruses

Viruses used in the studies were constructed following the manufacturer’s manual for the Bac-to-Bac expression system from Invitrogen. The pFastBac1 recombination vectors containing the cDNAs for all 11 wild-type CMG subunits were described in detail before (Ilves et al., 2010). These vector templates were used for generation of CMG mutants using PCR-based mutagenesis. Arginine finger residues were targeted and alanine substitutions were introduced to generate the RA point mutant. These include R641 in MCM2, R473 in MCM3, R645 in MCM4, R510 in MCM5, R521 in MCM6, and R514 in MCM7.

#### Protein Expression and Purification

##### Expression of *S. Cerevisiae* Proteins

All yeast proteins (except Ctf4) were expressed in *S. cerevisiae* cells and harvested following the same procedure. Cells were grown at 30°C in YEP media supplemented with 2% raffinose. At a cell density of ∼2-3x10^7^ cells/ml, expression was induced for 3 hours by the addition of 2% galactose. Cells were harvested by centrifugation at 5,020 x g for 30 min at 4°C. After washing pellets in lysis buffer (see individual protein purifications for buffer details) cells were re-centrifuged at 4,000 x g for 20 min at 4°C. Cells were subsequently resuspended in lysis buffer at half pellet volumes, flash frozen in liquid nitrogen and crushed at −80°C using a 6875D Freezer/Mill® Dual Chamber Cryogenic Grinderfreezer mill (SPEX SamplePrep) at intensity 15 (6 cycles of 2 min milling with 1 min rest).

##### Purification of Yeast CMG

*Sc*CMG was expressed and purified as previously described using the yeast strain yJCZ3 (Zhou et al., 2017). Following harvesting in CMG lysis buffer (25 mM HEPES pH 7.6, 15 mM KCl, 2 mM MgCl_2_, 0.02% Tween-20, 1 mM EDTA, 1 mM EGTA, 10% glycerol, 2 mM β-Mercaptoethanol, complete protease inhibitor tablets (Roche)) the cell powder was resuspended in Buffer C-100 (25 mM HEPES pH 7.6, 100 mM KCl, 0.02% Tween-20, 1 mM EDTA, 1 mM EGTA, 10% glycerol, 1 mM DTT) supplemented with 10 mM Mg(OAc)_2_, 25 units/ml benzonase (Sigma Aldrich) and complete protease inhibitor tablets (Roche). The lysate was incubated at 4°C for 45 minutes and cleared by ultracentrifugation at 235,000 x g for 60 minutes at 4°C. Clear supernatants were incubated for 3 hours at 4°C with 4 mL anti-FLAG M2 agarose beads (Sigma Aldrich) pre-equilibrated in Buffer C-100. Beads were subsequently washed with 150 mL Buffer C-100 after which bound proteins were eluted by incubation at room temperature for 30 minutes with the same buffer supplemented with 500 μg/ml FLAG peptide (DYKDDDDK) and complete protease inhibitor tablets (Roche). The eluate was collected and further proteins were eluted by repeating the FLAG peptide incubation for an additional 20 minutes. Combined eluates were passed through a 1 mL HiTrap SPFF column (GE Healthcare) and injected onto a MonoQ 5/50 GL column (GE Healthcare), both equilibrated in Buffer C-100. Proteins were washed with 10 CV of the same buffer and eluted with a 100-550 mM KCl gradient over 20 CV in Buffer C (25 mM HEPES pH 7.6, 0.02% Tween-20, 1 mM EDTA, 1 mM EGTA, 10% glycerol, 1 mM DTT). CMG peak fractions were diluted in Buffer C to 150 mM KCl and injected onto a MonoQ 1.6/5 PC column (GE Healthcare) equilibrated in Buffer C-150 (25 mM HEPES pH 7.6, 150 mM KCl, 0.02% Tween-20, 1 mM EDTA, 1 mM EGTA, 10% glycerol, 1 mM DTT). Proteins were washed with 10 CV of the same buffer and eluted with a 150-550 mM KCl gradient over 15 CV in Buffer C. CMG peak fractions were dialysed against Protein Binding Buffer (25 mM HEPES pH 7.6, 100 mM KOAc, 2 mM Mg(OAc)_2_, 5% glycerol, 0.02% NP-40, 1 mM DTT) for 3 hours at 4°C.

##### Purification of DNA Polymerase ε

*Sc*Polε was expressed and purified as previously described using the yeast strain yAE99 (Polε exo- mutant)(Goswami et al., 2018). Following harvesting in Buffer E-500 (25 mM HEPES pH 7.6, 400 mM KOAc, 10% glycerol, 1 mM DTT) supplemented with complete protease inhibitor tablets (Roche), the cell powder was resuspended in Buffer E-400 (25 mM HEPES pH 7.6, 400 mM KOAc, 10% glycerol, 1 mM DTT) supplemented with complete protease inhibitor tablets (Roche). The lysate was incubated at 4°C for 45 minutes and cleared by ultracentrifugation at 235,000 x g for 60 minutes at 4°C. Clear supernatants were supplemented with 2 mM CaCl_2_ and incubated for 2 hours at 4°C with 3 mL Calmodulin Affinity Resin (Agilent) pre-equilibrated in Buffer E-400. Beads were subsequently washed with 300 mL Buffer E-400 supplemented with 2 mM CaCl_2_ after which bound proteins were eluted by incubation at 4°C with Buffer E-400 supplemented with 2 mM EDTA and 2 mM EGTA. Pooled elutions were injected onto an SP Sepharose Fast Flow 1 mL column (GE Healthcare) attached to a MonoQ 5/50 GL column (GE Healthcare) and washed with 20 CV Buffer E-400. Following removal of the SP Sepharose Fast Flow column, proteins were eluted with a 400-1,000 mM KOAc gradient over 15 CV in Buffer E (25 mM HEPES pH 7.6, 10% glycerol, 1 mM DTT). Polε fractions were pooled, dialysed against Buffer E-400 and concentrated using a 30,000 MWCO cut-off spin column. 50 μl concentrated sample was subsequently passed over a Superose 6 3.2/300 gel filtration column in Buffer E-400.

##### Purification of Mrc1

*Sc*Mrc1 was expressed and purified as previously described using the yeast strain yJY32(Yeeles et al., 2017). Cells were harvested, lysed and resuspended in Buffer T-400 (25 mM Tris-HCl pH 7.5, 400 mM NaCl, 10% glycerol, 0.01% Tween-20, 1 mM DTT) supplemented with complete protease inhibitor tablets (Roche). The lysate was incubated at 4°C for 45 minutes and cleared by ultracentrifugation at 235,000 x g for 60 minutes at 4°C. Clear supernatants were incubated for 2 hours at 4°C with 2 mL anti-FLAG M2 agarose beads (Sigma Aldrich) pre-equilibrated in Buffer T-400. Beads were subsequently washed with 50 CV Buffer T-400 and 25 CV Buffer T-200 (25 mM Tris-HCl pH 7.5, 200 mM NaCl, 10% glycerol, 0.01% Tween-20, 1 mM DTT) followed by incubation for 10 min in Buffer T-200 supplemented with 1 mM ATP and 10 mM Mg(OAc)_2_. After washing beads in 10 CV Buffer T-200, bound proteins were eluted by incubation at room temperature for 45 minutes with the same buffer supplemented with 500 μg/ml FLAG peptide (DYKDDDDK) and complete protease inhibitor tablets (Roche). The eluate was collected and further proteins were eluted by repeating the FLAG peptide incubation for an additional 30 minutes. Combined eluates were subsequently injected onto a MonoQ 1.6/5 PC column (GE Healthcare) equilibrated in Buffer T-200. Proteins were washed with 10 CV of the same buffer and eluted with a 200-600 mM NaCl gradient over 15 CV in Buffer T (25 mM Tris-HCl pH 7.5, 10% glycerol, 0.01% Tween-20, 1 mM DTT). Mrc1 peak fractions were dialysed against Buffer T-200.

##### Purification of Csm3-Tof1

*Sc*Csm3/Tof1 was co-expressed and co-purified as previously described using the yeast strain yAE48(Yeeles et al., 2017). Cells were harvested, lysed and resuspended in CBP lysis buffer (25 mM Tris-HCl pH 7.5, 200 mM NaCl, 10% glycerol, 0.01% NP-40, 1 mM DTT) supplemented with complete protease inhibitor tablets (Roche). The lysate was incubated at 4°C for 45 minutes and cleared by ultracentrifugation at 235,000 x g for 60 minutes at 4°C. Clear supernatants were supplemented with 2 mM CaCl_2_ and incubated for 2 hours at 4°C with 2 mL Calmodulin Affinity Resin (Agilent) pre-equilibrated in CBP lysis buffer. Beads were subsequently washed with 75 CV CBP lysis buffer supplemented with 2 mM CaCl_2_ after which bound proteins were eluted by incubation at 4°C with CBP lysis buffer supplemented with 2 mM EDTA and 2 mM EGTA. Pooled elutions were concentrated to 500 μl using a 30,000 MWCO cut-off spin column and passed over a Superdex 200 10/300 gel filtration column equilibrated in CBP Gel Filtration Buffer (25 mM Tris-Hcl pH 7.5, 150 mM NaCl, 1 mM DTT). Csm3-Tof1 peak fractions were pooled and concentrated to 100 μl using a 30,000 MWCO cut-off spin column.

##### Expression and Purification of Ctf4 Trimer

*Sc*Ctf4 expression plasmids(Gambus et al., 2009) were transformed into BL21 (DE3) *E. coli* cells. Cells were grown in LB media at 37°C to an optical density (OD = 600) of 0.5 before expression was induced with 1 mM IPTG for 3 hours. Cells were harvested by centrifugation at 5,020 x g for 20 min at room temperature. Pelleted cells were subsequently resuspended in Ctf4 lysis buffer (50 mM Tris-HCl pH 7.5, 500 mM NaCl, 10 mM MgCl_2_, 10% glycerol, 1 mM DTT) supplemented with complete protease inhibitor tablets (Roche) and lysed by sonication. Lysate was cleared by centrifugation at 27,216 xg for 30 min at 4°C and incubated for 90 min at 4°C with 1 mL Ni-NTA Agarose Resin (QIAGEN) pre-equilibrated in Buffer A-20 (50 mM Tris-HCl pH 7.5, 300 mM NaCl, 20 mM imidazole). After washing resin with 20 CV Buffer A-20, proteins were eluted five times with 1 mL Buffer A-250 (50 mM Tris-HCl pH 7.5, 300 mM NaCl, 250 mM imidazole). Elutions were pooled and dialysed against Buffer B-100 (20 mM Tris-HCl pH 8.0, 100 mM NaCl, 1 mM DTT) before injection onto a MonoQ 5/50 GL column (GE Healthcare) equilibrated in Buffer B-100. Proteins were washed with 10 CV of the same buffer and eluted with a 100-1,000 mM NaCl gradient over 30 CV in Buffer B (20 mM Tris-HCl pH 8.0, 1 mM DTT). Ctf4 peak fractions were pooled and concentrated to 450 μl using a 30,000 MWCO cut-off spin column before being passed over a Superdex 200 16/600 gel filtration column equilibrated in Buffer B-150 (20 mM Tris-HCl pH 8.0, 150 mM NaCl, 1 mM DTT). Ctf4 trimer peak fractions were pooled and concentrated to 4 mg/ml using a 30,000 MWCO cut-off spin column.

##### Expression and Purification of *Drosophila Melanogaster* CMG

*Drosophila melanogaster* CMG was expressed and purified as previously described (Abid Ali et al., 2016, Ilves et al., 2010). Following bacmid generation for each subunit of *Dm*CMG, Sf21 cells were used for transfection and virus amplification stages to generate P2 stocks using serum-free Sf-900TM III SFM insect cell medium (Invitrogen/GIBCO). In the P3 virus amplification stage, 100 mL Sf9 cell (0.5x10^5^/ml) cultures were infected with 0.5 mL of P2 stocks with an approximate MOI of 0.1 for each virus and incubated in 500 mL Erlenmeyer sterile flasks (Corning) for 4 days at 27°C, shaking at 100 rpm. After 4 days, 4 L of Hi-Five cells (10^6^/ml) supplemented with 10% FCS were infected using fresh P3 stocks with MOI of 5. Cells were incubated at 27°C and harvested after 60 hours. Cell pellets were washed with PBS supplemented with 5 mM MgCl_2,_ resuspended in lysis buffer and frozen in 10 mL aliquots on dry ice. Protein purification was performed at 4°C. Cell pellets were thawed and lysed by applying at least 50 strokes per 30 mL of cell pellets using tissue grinders (Wheaton, 40 mL Dounce Tissue Grinder) after which the lysate was cleared by centrifugation at 24,000 x g for 10 min. Supernatants were incubated for 2.5 hours with 2 mL ANTI-FLAG M2 agarose beads (Sigma Aldrich) pre-equilibrated with Buffer C. Non-bound proteins were removed by centrifugation at 200 x g for 5 minutes followed by bead washing with 30 mL of Buffer C-100. Bound proteins were subsequently eluted by incubation at room temperature for 15 min with Buffer C-100 supplemented with 200 μg/ml FLAG peptide (DYKDDDDK). The eluate was passed through a 1 mL HiTrap SPFF column (GE Healthcare) and injected onto a MonoQ 5/50 GL column (GE Healthcare), both equilibrated in buffer C-100. Proteins were washed with 10 CV of the same buffer and eluted with a 100-550 mM KCl gradient over 20 CV in buffer C. CMG peak fractions were diluted in buffer C to 150 mM KCl and injected onto a MonoQ 1.6/5 PC column (GE Healthcare) equilibrated in buffer C-150. Proteins were washed with 10 CV of the same buffer and eluted with a 150-550 mM KCl gradient over 15 CV in Buffer C. CMG peak fractions were dialysed into Protein Binding Buffer (25 mM HEPES pH 7.6, 100 mM KOAc, 2 mM Mg(OAc)_2_, 5% glycerol, 0.02% NP-40, 1 mM DTT) for 2 hours.

#### Forked DNA Unwinding Assay

A list of oligonucleotides is provided in Table S3. Roadblock experiments. To prepare Cy5-labeled fork DNA substrate containing a single MH roadblock on the leading-strand template, oligonucleotides A, B and C were annealed at equimolar concentrations, and the resulting nick was sealed with T4 DNA ligase. DNA was purified via electroelution after separating on 8% PAGE. M.HpaII (NEB) was crosslinked in methyltransferase buffer (50 mM Tris-HCl pH 7.5, 0.5 mM 2-mercaptoethanol, 10 mM EDTA, NEB) supplemented with 100 μM S-adenosylmethionine (NEB), and incubated at 37°C for 3 hours. M.HpaII crosslinked substrate was separated on 8% PAGE and purified via electroelution.

Cy5-labeled fork DNA with a lagging-strand MH roadblock was prepared by annealing oligonucleotides D and E. The substrate was gel purified, crosslinked to MH, and re-purified as described above.

For unwinding assays, *Drosophila* CMG was first bound to fork DNA by incubating 3-5 nM DNA substrate with 30 nM *Drosophila* CMG in CMG-binding buffer (25 mM HEPES pH 7.5, 5 mM NaCl, 10 mM magnesium acetate, 5 mM DTT, 0.1 mg/ml BSA) supplemented with 0.1 mM ATPγS in 5 μL volume at 37°C for 2 hours. To initiate unwinding, 15 μL ATP mix (CMG-binding buffer with 3.3 mM ATP) was added into the reaction. The ATP mix contained 1.5 μM 40 nt polyT oligonucleotide to capture free CMG and 150 nM oligonucleotide with the sequence 5′-GGATGCTGAGGCAATGGGAATTCGCCAACC-3′ to prevent re-annealing of DNA. After further 30 min incubation at 37°C, reactions were stopped with SDS-containing buffer, separated on 8% PAGE, and imaged on Fujifilm SLA-5000 scanner using 635-nm laser and LPR/R665 filter.

#### M13-Based DNA Unwinding Assay

A 70-mer oligonucleotide (“T”) was designed such that 40 nucleotides anneal to a M13mp18ssDNA plasmid (New England Biolabs), leaving a 30-mer polyT extension at the 5′ end. The 5′ end was previously radioactively labeled with γ-^32^P ATP (MP Biomedicals or Perkin Elmer) and T4 polynucleotide kinase (New England Biolabs), subsequently purified through a llustra MicroSpin G-50 column (GE Healthcare) and mixed with the M13mp18 ssDNA plasmid. The reactions were heat-denatured for 1 minute and annealed through gradual cooling to room temperature. Free oligonucleotide was separated by purification through MicroSpin S-400 HR columns (GE Healthcare). The helicase assays were carried out in 25mM HEPES pH 7.6, 10% glycerol, 50mM sodium acetate, 10mM magnesium acetate, 0.2mM PMSF, 1mM DTT, with addition of 250 μg/ml insulin. Desired protein concentrations were mixed with 1-2fmol of a circular M13 based DNA substrate and unwinding initiated in the presence of 0.3mM ATP in a total reaction volume of 10 μL at 30°C. Reactions were stopped after 30 minutes by addition of 0.1% SDS and 20mM EDTA, and the reaction products were immediately electrophoretically separated on a TBE-acrylamide gel (8% TBE with 0.1%SDS).

#### Fork Affinity Purification of CMG

To prepare desthiobiotin-tagged, M.HpaII-labeled DNA fork substrates containing two M.HpaII on the leading-strand template, oligonucleotides F, G, H and I were annealed at a 1:1:2:1 molar ratio, and the resulting nicks were sealed with T4 DNA ligase. DNA was purified via electroelution after separating on 8% PAGE. M.HpaII (NEB) was crosslinked in methyltransferase buffer (50 mM Tris-HCl pH 7.5, 0.5 mM 2-mercaptoethanol, 10 mM EDTA, NEB) supplemented with 100 μM S-adenosylmethionine (NEB), and incubated at 37°C for 5 hours. M.HpaII crosslinked substrate was separated on 8% PAGE and purified via electroelution. To prepare desthiobiotin-tagged MH-labeled DNA fork substrates containing one MH on the leading-strand template and one M.HpaII on the lagging-strand template, oligonucleotides J + K and L + M were annealed separately at equimolar concentrations. The annealed oligonucleotide samples were then mixed and nicks were sealed with T4 DNA ligase. The substrate was gel purified, crosslinked to M.HpaII, and purified as described above. To isolate DNA-bound CMG complexes, a fork affinity purification approach was adapted from a previously published method(Goswami et al., 2018). Here, desthiobiotin-tagged DNA forks were immobilised onto streptavidin-coated magnetic beads. 6 μl M-280 Streptavidin Dynabeads® (Thermo Fisher) slurry was added to each reaction tube and washed twice in 20 μl DNA Binding Buffer (25 mM HEPES 7.6, 1M NaCl, 10% glycerol, 0.01% NP-40, 1 mM EDTA). Washed beads were resuspended in 20 μl 250 nM MH-conjugated DNA forks and incubated for 30 minutes at 30°C shaking at 1,250 rpm in a thermomixer. All subsequent incubations were performed at the same conditions. Following fork immobilisation, supernatants were discarded and beads were washed once in DNA Binding Buffer and once in Protein Binding Buffer. Fork-bound beads were subsequently resuspended in 250 nM CMG supplemented with 2 mM ATPγS and incubated for 30 minutes. Supernatants were collected to eliminate non-bound CMG and beads were washed twice in Protein Binding Buffer with 2 mM ATPγS (the second wash was performed without glycerol). CMG-bound DNA-forks were eluted from beads by resuspension in 10 μl Elution Buffer (25 mM HEPES pH 7.6, 100 mM KOAc, 2 mM Mg(OAc)_2_, 5% glycerol, 0.02% NP-40, 1 mM DTT, 400 nM biotin) supplemented with 2 mM ATPγS or 5 mM ATP followed by incubation for 30 minutes. The ATP elution with forks harboring both leading and lagging strand roadblocks was also supplemented by 1 μM of oligonucleotide Q. Supernatants were pooled and used for negative stain or cryo-EM grid preparation.

#### Fork Affinity Purification of CMG-Polε-Mrc1-Csm3-Tof1

To prepare desthiobiotin-tagged fork DNA substrates, oligonucleotides N and O were annealed at a 1:1.2 molar ratio and subsequently immobilised onto streptavidin-coated magnetic beads as described above. 250 nM CMG was mixed with 350 nM MCT (Mrc1 + Csm3-Tof1) in the presence of 2 mM ATPγS and incubated on ice for 5 min. Following washing in Protein Binding Buffer, fork-bound beads were resuspended in the CMG-MCT sample and incubated for 30 min before addition of 80 nM Polε and co-incubation for another 15 min. Protein-bound DNA-forks were washed twice in Protein Binding Buffer (the second wash was performed without glycerol) with 1 mM ATPγS and eluted from beads by resuspension in 12 μl Elution Buffer supplemented with 1 mM ATPγS or ATP. Supernatants were pooled and used for negative stain EM grid preparation. In a parallel experiment, the same affinity purification was performed in the absence of Polε using a DNA fork labeled with two leading strand M.HpaII-conjugates (same constructs used for “Fork affinity purification of CMG”).

#### DNA Affinity Purification of Mrc1-Csm3-Tof1

To prepare desthiobiotin-tagged duplex DNA substrates, oligonucleotide P was PCR-amplified using primers R and S. Desthiobiotin-tagged fork DNA substrates were prepared as in above CMG-Polε-Mrc1-Csm3-Tof1 affinity purification. 250 nM DNA constructs were immobilised onto streptavidin-coated magnetic beads as described above. Following washing in Protein Binding Buffer, fork-bound beads were resuspended in 20 μl 350 nM Mrc1, 350 nM Csm3-Tof1 or 350 nM MCT pre-incubated on ice for 5 min. Protein-DNA samples were incubated for 30 min at 30°C shaking at 1,250 rpm. Beads were washed twice in Protein Binding Buffer (the second wash was performed without glycerol) after which DNA-bound proteins were eluted by resuspension in 12 μl Elution Buffer.

#### Reconstitution of CMG-Ctf4

100 μl 250 nM CMG supplemented with 2 mM ATPγS was added to 100 μl Calmodulin Affinity Resin (Agilent) equilibrated in Protein Binding Buffer and incubated for 2 hours at 4°C. Beads were subsequently washed in 100 μl PBB with 1 mM ATPγS and resuspended in 100 μl 500 nM Ctf4 supplemented by 1 mM ATPγS. Following incubation for 30 min at 30°C shaking at 1,250 rpm the beads were washed twice in PBB with 1 mM ATPγS and resuspended in 50 μl CBP Elution Buffer (25 mM HEPES pH 7.5, 100 mM KOAc, 2 mM MgOAc, 5 mM EDTA, 5 mM EGTA, 0.01% Tween-20, 1 mM DTT) supplemented by 1 mM ATPγS. After incubation for an additional 30 minutes the supernatant was separated and incubated with 0.01% glutaraldehyde for 5 min. Cross-linked samples were immediately applied to EM grids for negative staining.

#### Reconstitution of CMG-Csm3-Tof1

100 μl 200 nM CMG supplemented with 2 mM ATPγS was added to 100 μl anti-FLAG M2 agarose beads (Sigma Aldrich) equilibrated in Protein Binding Buffer with 5 mM MgOAc and incubated for 2.5 hours at 4°C. Beads were subsequently washed in 450 μl PBB with 5 mM MgOAc and 1 mM ATPγS and resuspended in 100 μl 500 nM Csm3-Tof1 supplemented by 1 mM ATPγS. Following incubation for 30 min at 4°C shaking at 1,250 rpm the beads were washed twice in PBB with 5 mM MgOAc and 1 mM ATPγS and resuspended in 100 μl FLAG Elution Buffer (25 mM HEPES pH 7.5, 100 mM KOAc, 5 mM MgOAc, 0.01% Tween-20, 1 mM DTT, 2.5 mg/ml 3x FLAG peptide) supplemented by 1 mM ATPγS. After incubation for an additional 30 minutes at room temperature the supernatant was separated and applied to EM grids for negative staining.

#### Electrophoretic Mobility Shift Assay

Purified Mrc1 and/or Csm3-Tof1 was serially diluted in Protein Binding Buffer (200, 400, 800, 1600 and 3200 nM) and preincubated on ice for 5 min. Diluted samples were subsequently mixed with 300 nM duplex DNA or DNA forks (same DNA constructs used for *DNA affinity purification of Mrc1-Csm3-Tof1*) in 10 μl reactions and incubated for 30 min on ice. Protein-DNA complexes were resolved by polyacrylamide gel electrophoresis (PAGE) using an 4% polyacrylamide gel ran at 100 V for 90 min (4°C) in 0.5x TAE buffer after which nucleic acids were visualized by staining with SYBR Safe. To verify that the DNA binding function is contained in Csm3-Tof1 and Mrc1 and not in uncharacterized contaminant proteins, we performed native PAGE super-shift assays using antibodies specific for the CBP-Csm3 or the FLAG-Mrc1. DNA-Csm3-Tof1 complexes were pre-assembled by mixing Csm3-Tof1 and DNA-fork substate at a concentration of 200nM and 300nm respectively. DNA-Mrc1 complexes were pre-assembled by mixing Mrc1 and fork at a concentration of 1600nM and 300nM respectively. The pre-assembled complexes were mixed with anti-CBP antibody (SIGMA 07-4820) or anti-FLAG antibody (SIGMA F3165-.2MG, prepared according to manufacturer’s instruction) to respectively assay DNA-Csm3-Tof1 or DNA-Mrc1 complex formation. The DNA-Csm3-Tof1 anti-CBP super-shift was resolved using a 4% PAGE run in 0.5x TAE. The DNA-Mrc1 anti-FLAG super-shift was resolved using a 1.2% agarose gel, run in 0.2x TB. Both gels were stained with SYBR Safe.

#### EM Grid Preparation

##### Negative Stain EM Grids

300-mesh copper grids with a continuous carbon film (EM Resolutions, C300Cu100) were glow-discharged for 30 s at 45 mA with a 100x glow discharger (EMS). 4-μl samples were applied to glow-discharged grids and incubated for 1 minute. Following blotting of excess sample, grids were stained by stirring in four 75-μl drops of 2% uranyl acetate for 5, 10, 15 and 20 s respectively. Excess stain was subsequently blotted dry.

##### Cryo-EM Grids

400-mesh lacey grids with a layer of ultra-thin carbon (Agar Scientific) were glow-discharged for 1 min at 45 mA with a 100x glow discharger (EMS). 4-μl fork-bound *Dm*CMG eluted with ATP was applied to glow-discharged grids and incubated for 2 minutes. Excess sample was subsequently blotted away for 0.5 s using a Vitrobot Mark IV (FEI ThermoFisher) at 4°C and ∼90% humidity. To increase particle concentration, a second 4-μl sample was applied to blotted grids and incubated for 2 minutes. Following blotting for 3 s the sample was plunge-frozen into liquid ethane.

#### EM Data Collection

##### Negative Stain EM

Data were acquired on a FEI Tecnai LaB6 G2 Spirit electron microscope operated at 120kV and equipped with a 2K x 2K GATAN UltraScan 1000 CCD camera. Micrographs were collected at x30,000 nominal magnification (3.45 Å pixel size) with a defocus range of −0.5 to −2.5 μm.

##### Cryo-EM

High-resolution cryo-EM data were acquired on a Titan Krios operated at 300kV and equipped with a K2 Summit detector operated in counting mode with 30 frames per movie. Micrographs were collected at x130,000 nominal magnification (1.08 Å pixel size) using a total electron dose of 50 e/Å^2^ and a defocus range of −2.0 to −4.1 μm (see Table S1 for further details).

#### Image Processing

##### Negative Stain EM Image Processing

All particles were picked semi-automatically using e2boxer in EMAN2 v2.07 (Tang et al., 2007) and contrast transfer function parameters were estimated by Gctf v1.18 (Zhang, 2016). All further image processing was performed in RELION v2.1 (Fernandez-Leiro and Scheres, 2017, Kimanius et al., 2016). Particles were extracted with a box size of 128 pixels for initial reference-free 2D classification and CTF was corrected using the additional argument–only_flip_phases.

To allow visualization of roadblocks in fork affinity purified CMG samples, helicase side views were selected for further rounds of 2D classification following particle re-extraction using a larger (192-pixel) box size. *Sc*CMG samples (double leading strand M.HpaII) eluted with ATPγS or ATP showed 5,558 and 3,933 side-view particles respectively, out of which 587 (10.6%) and 468 (11.9%) displayed roadblock densities. Similarly, *Dm*CMG samples (double leading strand M.HpaII) eluted with ATPγS or ATP showed 4,875 and 31,485 side-view particles respectively, out of which 1,712 (35.1%) and 8,227 (26.1%) displayed additional roadblock densities. *Dm*CMG samples with leading and lagging strand M.HpaII roadblocks eluted with ATPγS or ATP showed 16,420 and 25,397 side-view particles respectively, out of which 820 (5.0%) and 4,162 (16.4%) displayed additional roadblock densities.

##### Cryo-EM Image Processing

The 30-frame movies collected were corrected for beam-induced motion using 5 × 5 patch alignment in MotionCor2 (Zheng et al., 2017) whereby all frames were integrated. CTF parameters were estimated on non dose-weighted micrographs by Gctf v.1.18 (Zhang, 2016). Particles were picked using crYOLO of the SPHIRE software package (Moriya et al., 2017). All subsequent image processing was performed in RELION-3 (Zivanov et al., 2018) and cryoSPARC (Punjani et al., 2017). An initial dataset of 3,296,333 binned-by-3 particles were extracted from 19,097 dose-weighted micrographs with a box size of 128 pixels (3.24 Å/pixel). After two rounds of 2D classification 1,151,231 high-resolution CMG averages were selected and re-extracted as unbinned particles with a box size of 384 pixels (1.08 Å/pixel). An initial 3D structure was generated by homogeneous refinement in cryoSPARC using a previous structure of DNA-bound CMG low-pass filtered to 30 Å as a starting model. The resulting CMG structure was subjected to three-dimensional classification with alignment in RELION that yielded 2 high-resolution classes with different DNA-binding modes in the central MCM channel. The remaining structures appeared severely anisotropic and were discarded.

The largest of the two structures after initial 3D classification (370,005 particles) was 3D refined in RELION followed by Bayesian particle polishing and one round of CTF refinement to solve a structure at 3.46 Å resolution (State 1). To better resolve DNA densities in the MCM central channel, ATPase domains were subtracted and the resulting particles were analyzed by 3D classification in RELION. In parallel efforts, focused 3D classification was performed on the ATPase domain of State 1 unsubtracted particles. These endeavors resulted in the identification of two states with a one-subunit register shift. 3D refinement, followed by Bayesian particle polishing and one round of CTF refinement of these structures allowed us to solve two structures at 3.70 Å (State 1A: 170,329 particles) and 3.99 Å (State 1B: 92,754 particles) resolution respectively.

The smaller of the two structures (State 2) after initial 3D classification (241,490 particles) was 3D refined in RELION and subjected to one additional round of 3D classification with alignment that eliminated some residual anisotropy and led to the determination of a structure from 117,560 particles. Following 3D refinement, Bayesian particle polishing and two rounds of CTF refinement, this structure was solved to 4.23 Å resolution (State 2). Further 3D classification of this particle subset, focused on the AAA+ domain, resulted in the identification of two structures with DNA-binding register shifted by one subunit. 3D refinement, Bayesian particle polishing and one round of CTF refinement of these structures allowed us to refine two structures at 4.28 Å (State 2A: 52,214 particles) and 4.46 Å (State 2B: 61,082 particles) resolution respectively.

An alternative initial 3D structure was generated by homogeneous refinement in cryoSPARC following less stringent 2D classification (2,251,730 particles). Further processing of this particle subset, including two rounds of heterogeneous refinement in cryoSPARC, allowed us to determine an alternative structure at 3.88 Å resolution (State 1^∗^: 152,519 particles) with ATPase DNA-binding similar to that of State 1, but with lagging strand density projecting from the N-terminal side of the helicase.

#### Model Building and Refinement

Homology models for *Drosophila* CMG were obtained using Swiss-Model (Waterhouse et al., 2018). The cryo-EM maps generated with RELION (Zivanov et al., 2018) were sharpened with phenix.auto_sharpen using resolution rage between 3.3 and 6 Å. To handle residual anisotropy in the structures three flags were employed, local_sharpening; local_aniso_in_local_sharpening and remove_aniso. While homology models for GINS and Cdc45 were initially docked as rigid bodies, MCM subunits were first split in three rigid bodies (A domain, B-C domains and AAA+ domain and simultaneously fitted into the cryo-EM density for each distinct state. The atomic models were subsequently refined using phenix.real_space_refine (Adams et al., 2010) with restrains for secondary structure elements and for planarity in the base pairing. The atomic models were corrected with Coot (Emsley et al., 2010) according to map density, geometries and chemistry. ATP and ADP molecules were manually fitted into densities. Single-stranded DNA was built by hand following the phosphate backbone and bases densities in Coot. The final atomic models were refined using phenix.real_space_refine with restrains for secondary structure elements and for base pair planarity. The quality of the atomic models was evaluated with the comprehensive cryo-EM validation tool in Phenix using the atomic models corrected with Coot and the maps generated by Relion Refine3D, as recommended in Afonine et al. (2018). Inter-protomer buried area was measured using the PDBe-PISA webserver (http://www.ebi.ac.uk/pdbe/pisa/), between each pair of neighboring MCM AAA+ domains or between each nucleotide and the opposed, Arg-finger providing ATPase module.

### Quantification and Statistical Analysis

Quantification, statistical analysis and validation pertaining to processing of negative stain and cryo-EM images are implemented in the software described in the image processing section of the methods details. Global resolution stimation of refined cryo-EM maps are based on the 0.143 cutoffs of the Fourier Shell Correlation between two half maps refined independently.

### Data and Code Availability

CMG-DNA maps and atomic models have been deposited with the Electron Microscopy Data Bank (EMDB) and the Protein Data Bank (PDB) under the following accession codes: State 1A, EMD-4785, PDB 6RAW; State 1B, EMD-4786, PDB 6RAX; State 2A, EMD-4787, PDB 6RAY; State 2B, EMD-4788, PDB 6RAZ. A reporting summary for this article is available in Supplementary Information.

### Additional Resources

We have not generated a new website or forum.

## Supplemental Information

Document S1. Figures S1–S7 and Tables S1–S3

Document S2. Article plus Supplemental Information
