# Supplementary material for: Molecular Basis for ATP-Hydrolysis-Driven DNA Translocation by the CMG Helicase of the Eukaryotic Replisome
Source: Cell Rep. 2019 Sep 3;28(10):2673–2688.e8. doi: 10.1016/j.celrep.2019.07.104 (PMC6737378; doi:10.1016/j.celrep.2019.07.104)
Supplement: Document S1. Figures S1–S7 and Tables S1–S3 [file mmc1.pdf]

Cell Reports, Volume *28*

Supplemental Information

Molecular Basis for ATP-Hydrolysis-Driven

DNA Translocation by the CMG Helicase

# of the Eukaryotic Replisome

Patrik Eickhoff, Hazal B. Kose, Fabrizio Martino, Tatjana Petojevic, Ferdos Abid Ali, Julia Locke, Nele Tamberg, Andrea Nans, James M. Berger, Michael R. Botchan, Hasan Yardimci, and Alessandro Costa

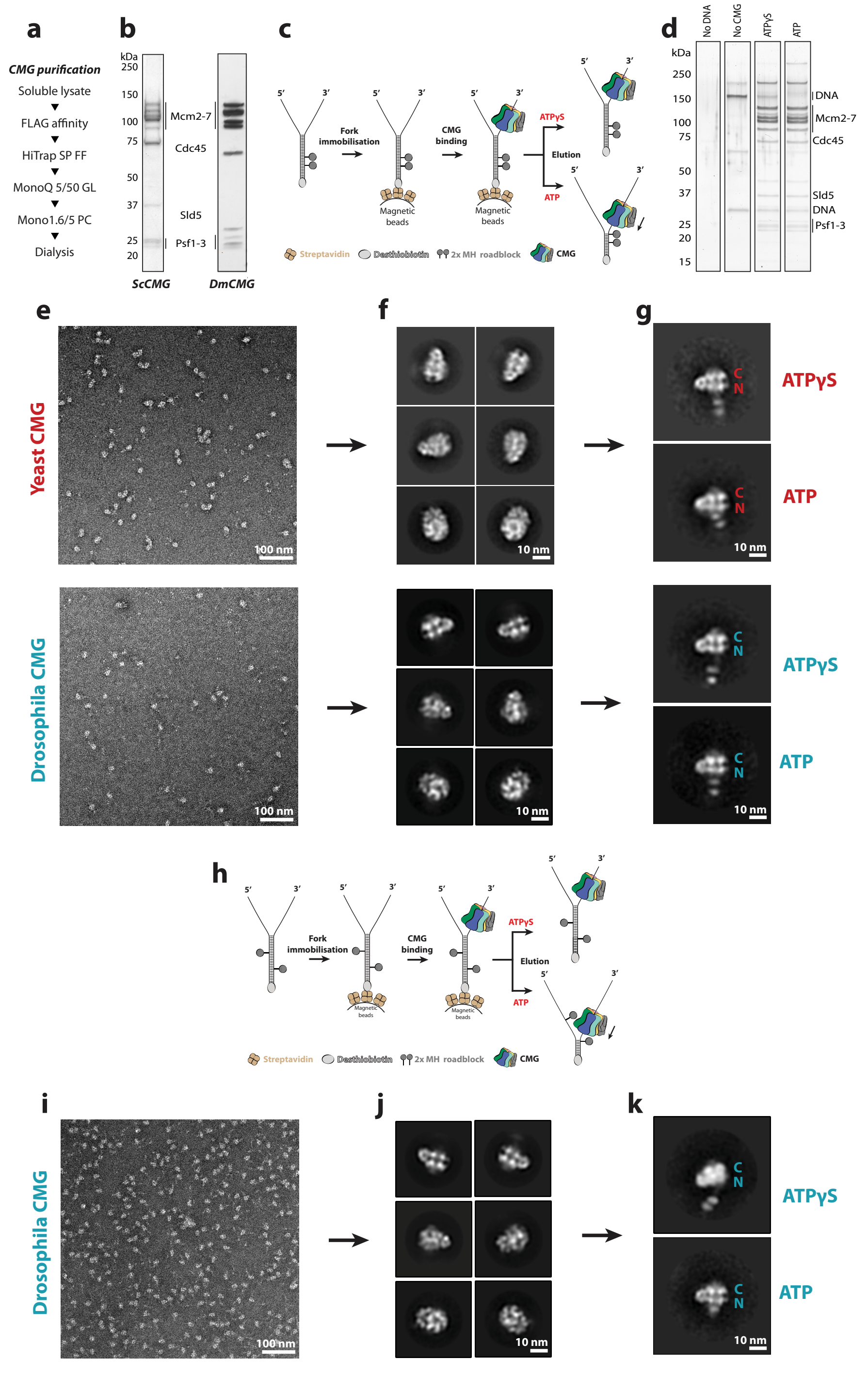

Figure S1 related to Figure 1: Isolation of the translocating CMG helicase. **(a)** CMG purification scheme. **(b)** Silver-stain gel of purified yeast and Drosophila CMG. For yeast CMG, mass spectrometry analysis identified non-CMG bands corresponding to endogenous Ctf4 and Pol epsilon components. **(c)** DNA-affinity purification scheme. Two HpaII Methyltransferase (MH) roadblocks are positioned on the leading strand. Elution is performed with biotin in buffer containing either ATPγS or ATP. **(d)** Nucleotide identity does not affect the yield of CMG-DNA elution as observed in silver-stain gel. Traces of endogenous Ctf4 and Pol epsilon can be visualized in the yeast CMG preparation, as established by mass spectrometry analysis. **(e)** Micrographs of yeast or Drosophila CMG-DNA. **(f)** One round of 2D classification yields CMG particles in various orientations. **(g)** Side-view sub-classification yields

classes with recognizable MH roadblocks. **(h)** DNA purification scheme with one MH roadblock on the lagging strand upstream of a leading strand roadblock. **(i)** Micrograph of *Dm*CMG-DNA with lagging/leading MH roadblocks. **(j)** One round of 2D classification yields CMG particles in various orientations. **(k)** Side-view sub-classification yields classes with recognizable MH roadblocks. In ATP buffer CMG bypasses the lagging-strand roadblock but not the leading-strand roadblock.

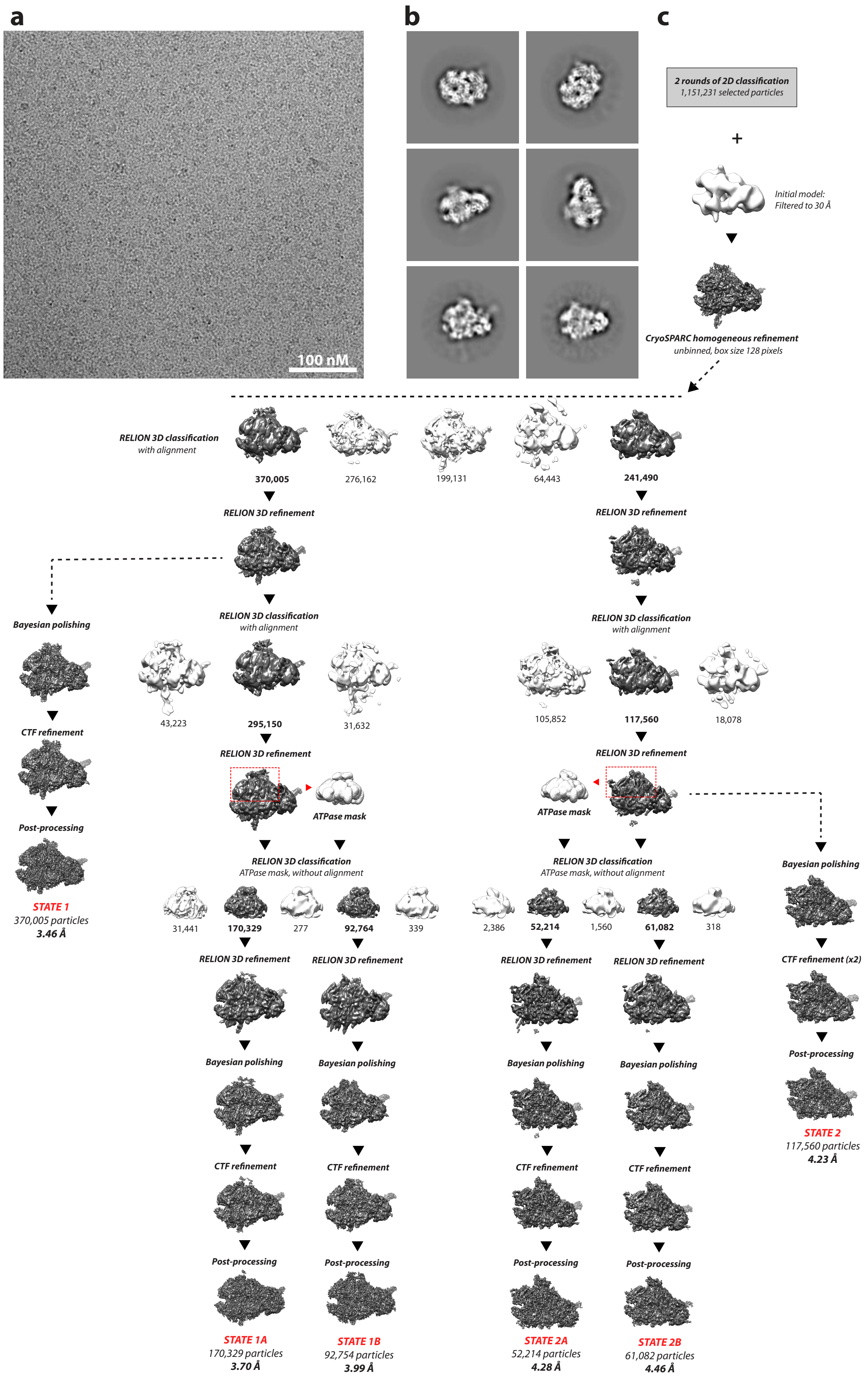

Figure S2 related to Figures 2, 3, 4 and 5: Cryo-EM of *Drosophila* CMG bound to MH-DNA. **(a)** Cryo-electron micrograph on a thin layer of continuous carbon. **(b)**

Representative 2D averages. **(c)** Summary of 3D classification and refinement performed with RELION.

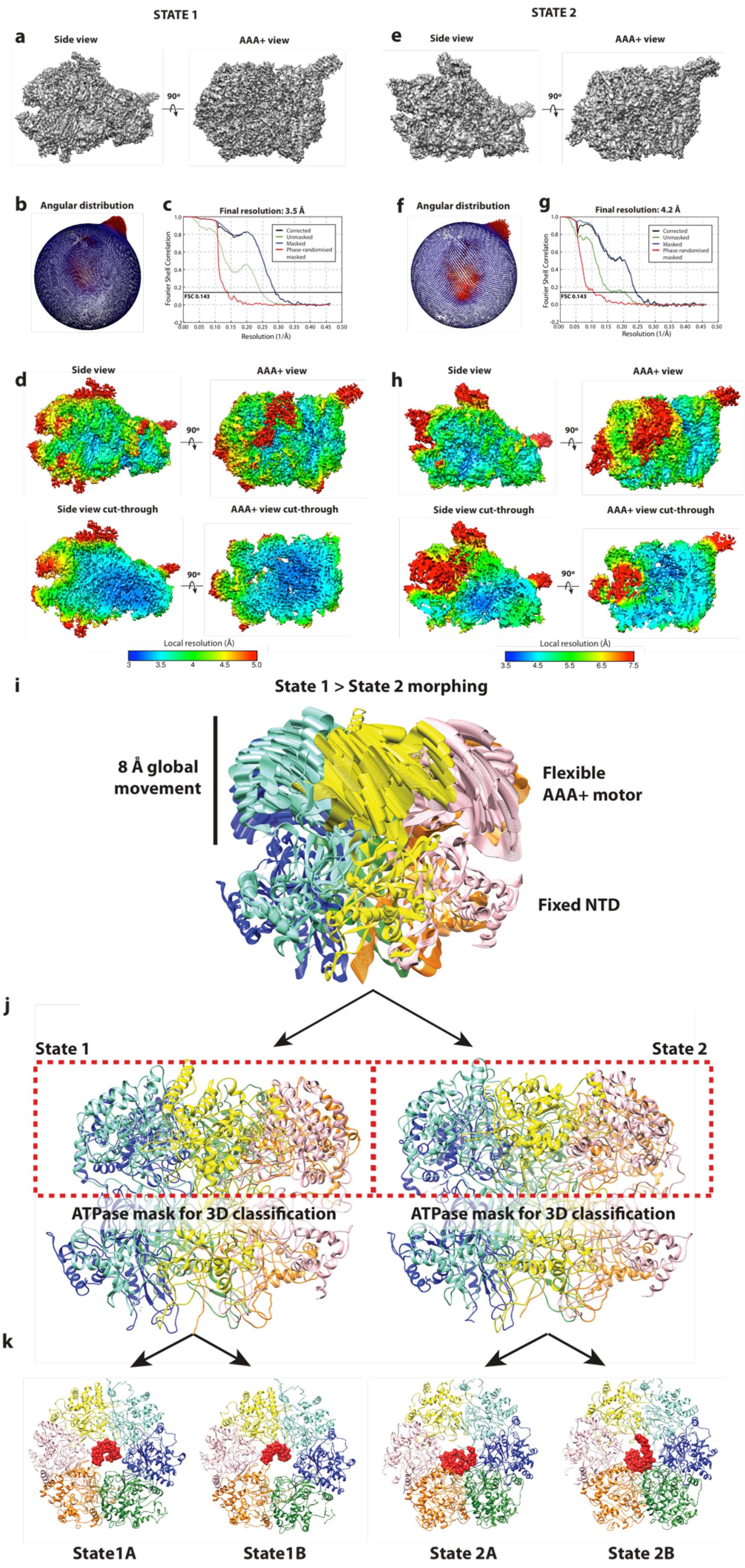
STATE 1
STATE 2
a
Side view
AAA+ view
90º
e
b
Angular distribution
c
Final resolution: 3.5 Å
Corrected
Unmasked
Masked
Phase-randomised masked
FSC 0.143
Fourier Shell Correlation
Resolution (1/Å)
f
g
Final resolution: 4.2 Å
d
h
Side view cut-through
AAA+ view cut-through
Local resolution (Å)
i
State 1 > State 2 morphing
8 Å global movement
Flexible AAA+ motor
Fixed NTD
j
State 1
State 2
ATPase mask for 3D classification
k
State1A
State1B
State 2A
State 2B

Figure S3 related to Figures 2, 3, 4 and 5: 3D refinement of CMG-DNA State 1 and State 2. State 1 **(a)** surface rendering, **(b)** angular distribution, **(c)** Fourier shell correlation and **(d)** local resolution map. State 2 **(e)** surface rendering, **(f)** angular distribution, **(g)** Fourier shell correlation and **(h)** local resolution map. (**i**) Rationale for focused ATPase domain 3D classification. CMG State 1 and State 2 were aligned by the N-terminal domain. Morphing between the two states highlighted an 8 Å resolution global movement of the AAA+ domain. **(j)** To identify different roational states within State 1 and State 2, the AAA+ tier was masked in separate 3D classification runs. **(k)** These efforts led to the identification of four distinct states with different DNA occupancy around the ATPase ring.

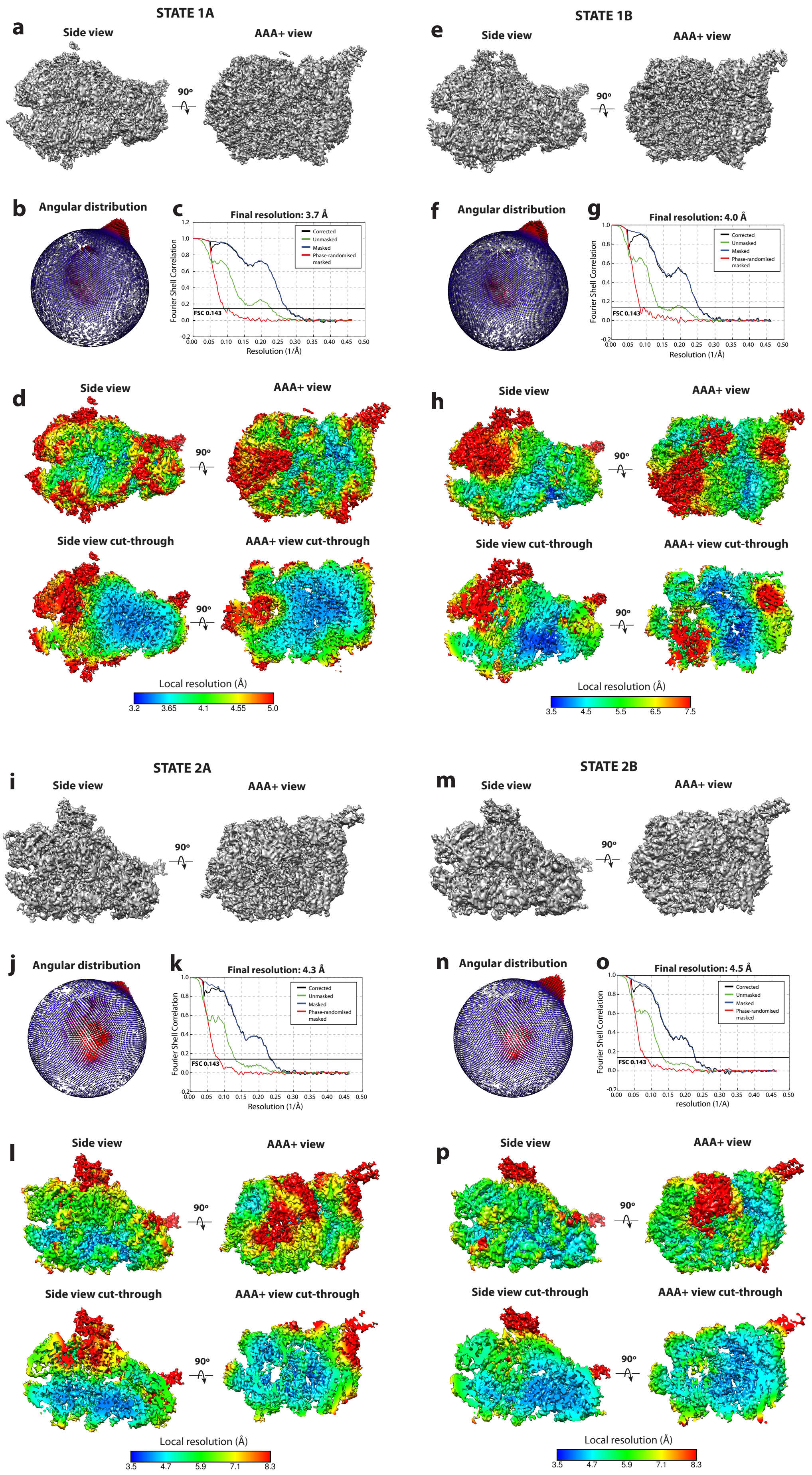
STATE 1A
a
Side view
AAA+ view
90º
b
Angular distribution
c
Final resolution: 3.7 Å
Fourier Shell Correlation
Resolution (1/Å)
Corrected
Unmasked
Masked
Phase-randomised masked
FSC 0.143
d
Side view
AAA+ view
Side view cut-through
AAA+ view cut-through
Local resolution (Å)
3.2
3.65
4.1
4.55
5.0
STATE 1B
e
f
Angular distribution
g
Final resolution: 4.0 Å
h
Local resolution (Å)
3.5
4.5
5.5
6.5
7.5
STATE 2A
i
j
k
Final resolution: 4.3 Å
l
Local resolution (Å)
3.5
4.7
5.9
7.1
8.3
STATE 2B
m
n
o
Final resolution: 4.5 Å
resolution (1/Å)
p

Figure S4 related to Figures 2, 3, 4 and 5: 3D refinement of CMG-DNA State 1A, State 1B, State 2A and State 2B. State 1A **(a)** surface rendering, **(b)** angular distribution, **(c)** Fourier shell correlation and **(d)** local resolution map. State 1B **(e)** surface rendering, **(f)** angular distribution, **(g)** Fourier shell correlation and **(h)** local resolution map. State 2A **(i)** surface rendering, **(j)** angular distribution, **(k)** Fourier shell correlation and **(l)** local resolution map. State 2B **(m)** surface rendering, **(n)** angular distribution, **(o)** Fourier shell correlation and **(p)** local resolution map.

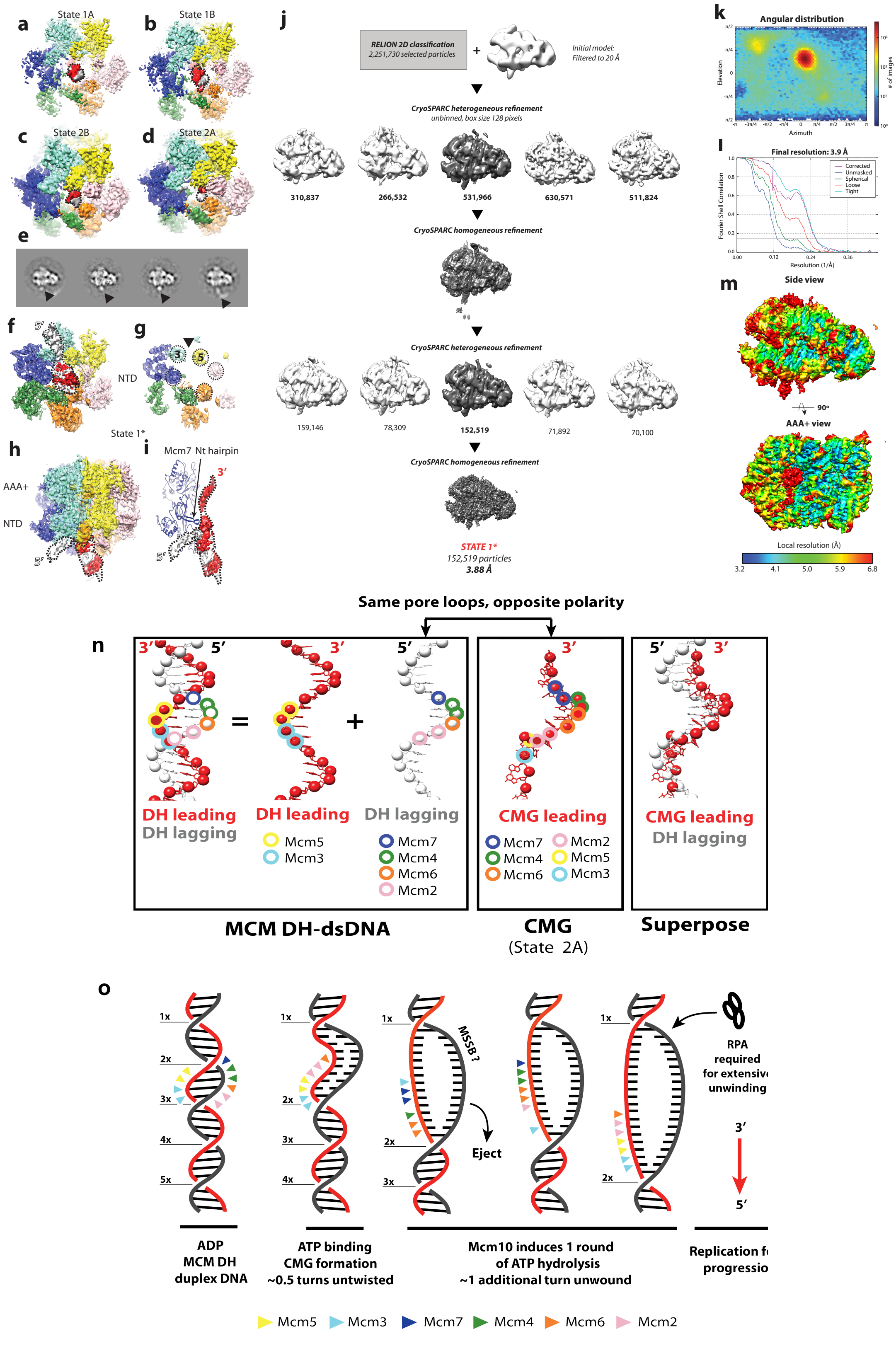

Figure S5 related to Figure 3 and Figure 7: Various CMG states interact with duplex DNA differently. **(a-b)** In state 1A and 1B a prominent duplex DNA density is visibly interacting with N-terminal MCM, in particular with the B

domains of Mcm5, 6 and 4. **(c)** In state 2B, limited duplex DNA density can be observed interacting with Mcm5 and Mcm6. **(d)** Duplex DNA density in State 2A is very poor/noisy. DNA densities were Gaussian-filtered in Chimera to aid visualization. Red desities represent the leading strand template and grey desities represent the lagging strand template. **(e)** To ensure that CMG State 2 particles engage the fork with the N-terminal MCM domain facing the fork nexus, we performed extensive 2D classification, which reveals a flexible density compatible with duplex DNA (and perhaps MH roadblock), proximal to the N-terminal MCM tier. This density is shown with a black arrowhead. **(f)** CryoSPARC-refined State 1* reveals a lagging-strand DNA path. Duplex DNA enters the MCM pore by the N-terminal tier where the lagging strand template (white) emerges from an opening between Mcm3 (cyan) and Mcm5 (yellow). **(g)** MCM B domains highlighted with dotted black circles. A black arrowhead points at an opening between Mcm3 and Mcm5. The particle orientation is identical to **(f)**, however DNA and posterior CMG densities are removed to highlight the B domains. **(h)** Side view of the same cryo-EM structure view in (f). **(i)** The Mcm7 N-terminal hairpin splits the leading (red) and lagging (white) strand templates at the fork nexus. **(j)** Heterogeneous and homogeneous refinement using cryoSPARC. CMG State 1* **(k)** angular distribution, **(l)** Fourier shell correlation and **(m)** local resolution map. **(n)** A mechanism for ATP-hydrolysis driven origin DNA melting by the CMG. Comparison between DNA contacts of the MCM double hexamer and the CMG in State 2A. In the double hexamer, Mcm3 and Mcm5 AAA+ modules touch the leading strand template, while Mcm2467 touch the lagging strand template. In CMG state 2A, ATPase pore loops of all six MCM subunits touch the leading strand only. Overlaying the MCM double hexamer and CMG in State 2A shows that the DNA contacts of the two states are very similar, although the same contacts engage lagging and leading strand respectively. Promiscuity in DNA binding by ATPase pore loops underlies the mechanism of duplex DNA melting by the CMG. **(o)** Cartoon representation of ATP-hydrolysis driven origin DNA melting. Transition from ADP-bound MCM double hexamer to ATP-bound CMG causes untwisting of half a turn of the double helix. At this stage, Mcm2,6,4 pore loops switch from contacting the lagging to the leading strand template. Mcm10 induces ATP hydrolysis, causing single-stranded DNA rotation around the MCM ring. During this process, all elements known to engage the lagging strand in the double hexamer concomitantly bind the leading strand template. The lagging strand is likely to visit a high energy state that causes the strand to be ejected from the helicase pore as soon as an opening is created in the MCM ring,

possibly by direct binding of Mcm10. The N-terminal MCM single-strended binding MSSB might have a role in mediating this transition. The end result of origin melting is unwinding of one additional turn of the double helix. Recruitment of Replication Protein A (RPA) would subsequently facilitate extensive DNA unwinding.

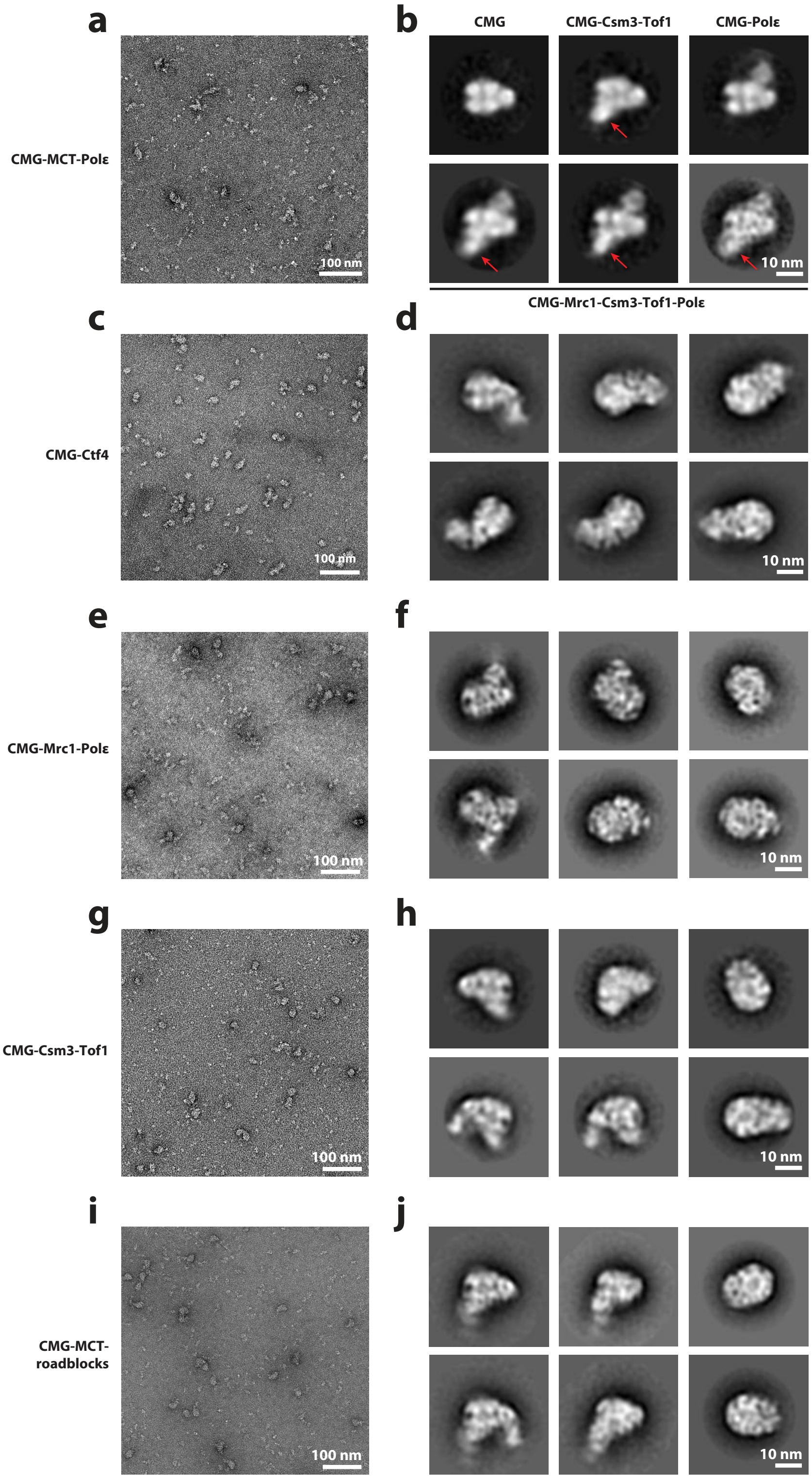

Figure S6 related to Figure 6**:** Negative stain EM imaging of various replisome complexes. **(a)** Negative-stain electron micrograph and **(b)** 2D averages of CMG-DNA-Pol epsilon-Csm3-Tof1-Mrc1. A number of distinct complexes coexist in the same dataset, containing DNA bound to CMG, CMG-Csm3-Tof1, CMG-Pol epsilon and the full CMG-Pol epsilon-Mrc1-Csm3-Tof1 complex. **(c)** Negative stain electron micrograph of reconstituted CMG-Ctf4 and **(d)** corresponding 2D averages.

Importantly, the Ctf4 density maps close to GINS and not in proximity of MCM where Csm3-Tof1 is located in the CMG-Pol epsilon-Mrc1-Csm3-Tof1 complex. **(e)** Negative-stain electron micrograph and **(f)** 2D averages of CMG-Pol epsilon-Mrc1.

The 2D class on the top right also contains a density for co-purified endogenous Ctf4. **(g)** Negative stain electron micrograph of reconstituted CMG-Csm3-Tof1 and **(h)** corresponding 2D averages. **(i)** Negative stain electron micrograph of reconstiuted CMG-MTC bound ATPγS and DNA containing two MH roadblocks, and **(j)** corresponding 2D averages.

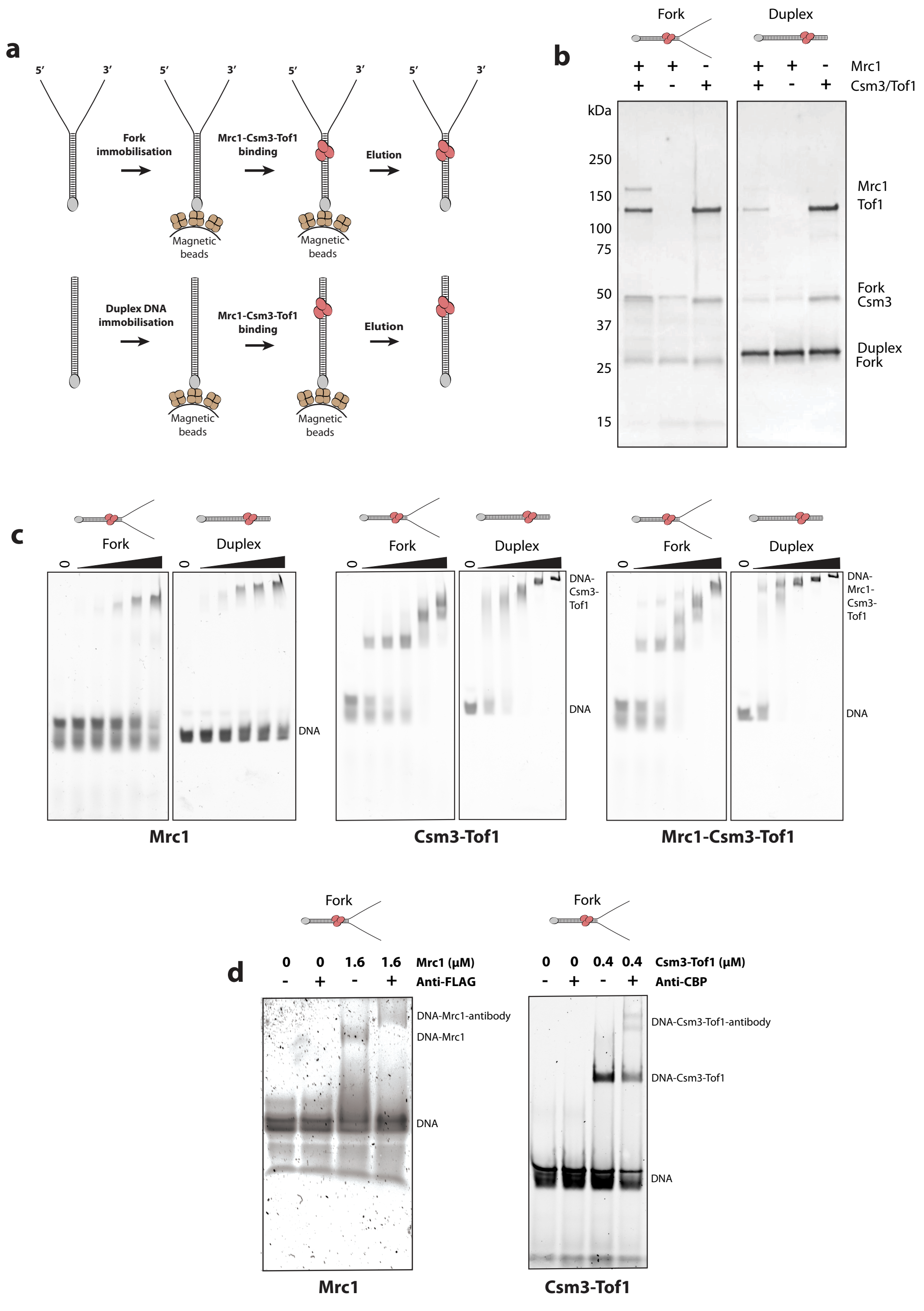

Figure S7 related to Figure 6**:** The MTC complex interacts with duplex DNA. **(a)** Schematic of DNA affinity purification of the MTC complex using either forked or blunt duplex DNA. **(b)** Silver-stained gel of fork affinity purified MTC indicates that Csm3-Tof1 are the major DNA binding components in the MTC complex. The complex binds forked DNA as well as blunt duplex DNA. **(c)** Electrophoretic mobility shift assays of the isolated Mrc1, Csm3-Tof1 or of the full MTC complex binding either forked or duplex DNA. Proteins were added at concentrations ranging from 0-

3200 nM (0, 200, 400, 800, 1600 or 3200 nM) to 300 nM DNA. Mrc1 is capable of binding both forked as well as blunt duplex DNA, although with lower affinity compared to Csm3-Tof1. The MTC complex shows the highest affinity for DNA. **(d)** Antibody-induced super-shift indicates that DNA binding is due to Mrc1 and Csm3-Tof1 and not due to uncharacterized contaminants. Co-incubation with anti-FLAG and anti-CBP antibody shows a clear supershift in electrophoretic mobility shift assays, indicating that DNA binding is dependent on FLAG-Mrc1 and CBP-Csm3/Tof1 respectively.

DmCMG cryo-EM data collection / processing				
**Structure**	**State 1A**	**State 1B**	**State 2A**	**State 2B**
**Microscope**	Titan Krios TEM (Thermo Scientific)			
**Detector**	K2 Summit (GATAN) in counting mode			
**Nominal magnification**	130000			
**Voltage (kV)**	300			
**Electron exposure ($e^-/Å^2$)**	50			
**Defocus range (μm)**	-2.0 to -4.1			
**Pixel size (Å)**	1.08			
**Symmetry**	C1			
**Initial particle images (no.)**	3296333			
**Final Particle images (no.)**	170329	92754	52214	61082
**Sharpening B-factor**	-70	-80	-70	-70
**FSC threshold**	0.143	0.143	0.143	0.143
**Map resolution (Å)**	3.7	3.99	4.28	4.46
**Map resolution range (Å)**	3.2-5.0	3.5-7.5	3.5-8.3	3.5-8.3
Atomic model refinement				
**Refinement software**	Phenix.real_space_refine	Phenix.real_space_refine	Phenix.real_space_refine	Phenix.real_space_refine
**Homology-modelling software**	SWISS-MODEL	SWISS-MODEL	SWISS-MODEL	SWISS-MODEL
**Initial models used (PDB code)**	6F0L (MCMs)<br>3JC6 (GINS / Cdc45)	6F0L (MCMs)<br>3JC6 (GINS / Cdc45)	6F0L (MCMs)<br>3JC6 (GINS / Cdc45)	6F0L (MCMs)<br>3JC6 (GINS / Cdc45)
**Map sharpening**	No	No	No	No
**Model composition**<br>Non-hydrogen atoms<br>Protein residues<br>Nucleotide<br>Ligands<br>Water	39773<br>4884<br>39<br>ATP: 3, ADP: 3<br>0	41066<br>5075<br>29<br>ATP: 3, ADP: 3<br>0	39193<br>4904<br>13<br>ATP: 4, ADP: 2<br>0	38333<br>29<br>ATP: 4, ADP: 2<br>0
***B* factors (Å2)**<br>AverageADP (B-factors)<br>Protein<br>Nucleotide<br>Ligand<br>Water	min / max / mean<br>32.41 / 343.42 / 128.01<br>96.76 / 599.89 / 419.59<br>67.18 / 135.31 / 97.39<br>-	min / max / mean<br>37.22 / 482.98 / 145.57<br>138.16 / 794.11 / 440.62<br>96.69 / 197.90 / 133.62<br>-	min / max / mean<br>30.00 / 825.82 / 209.3<br>321.05 / 818.61 / 529.54<br>102.83 / 245.63 / 180.07<br>-	min / max / mean<br>30.00 / 456.07 / 186.64<br>205.22 / 985.45 / 538.78<br>135.89 / 265.04 / 198.21<br>-
**R.m.s. deviations**<br>Bond lengths (Å)<br>Bond angles (°)	0.0008<br>1.266	0.012<br>1.448	0.013<br>1.749	0.011<br>1.541
**Validation**<br>MolProbity score<br>Clashscore<br>Rotamers outliers (%)<br>Cb outliers (%)	3.07<br>17.07<br>6.39<br>0.41	3.23<br>23.87<br>6.73<br>0.56	3.17<br>19.63<br>7.49<br>0.84	3.14<br>21.87<br>5.99<br>0.64
**Ramachandran plot**<br>Favored (%)<br>Allowed (%)<br>Outlier (%)	80.8<br>18.89<br>0.31	80.22<br>19.3<br>0.48	81.69<br>17.48<br>0.83	81.33<br>17.75<br>0.92
**Resolution Estimates (Å)**<br>d FSC model (0.143-0.5)	Masked<br>3.6 / 4.5	Masked<br>3.5 / 6.7	Masked<br>4.1 / 7.2	Masked<br>4.1/ 6.8
**CC (mask)**<br>**CC (box)**<br>**CC (volume)**<br>**Mean CC for ligands**	0.66<br>0.63<br>0.65<br>0.72	0.64<br>0.67<br>0.63<br>0.73	0.66<br>0.65<br>0.65<br>0.71	0.69<br>0.71<br>0.68<br>0.75

Table S1 related to Figure 2, 3, 4, 5: cryo-EM data collection, refinement and validation statistics.

STATE 1A	Mcm4>6 ADP	Mcm6>2 ATP	Mcm2>5 ATP	Mcm5>3 ATP	Mcm3>7 ADP	Mcm7>4 ADP
R finger β Ca > WA K β Ca (Å)	14.2	15.6	12.0	13.0	12.0	14.0
R finger β Ca > S3 H β Ca (Å)	15.0	14.9	12.8	14	11	15
Inter-AAA+ surface ($Å^2$)	1414	1620	2065	1900	1604	793
Inter-active site surface ($Å^2$)	155	220	209	237	158	106

STATE 1B	Mcm4>6 ADP	Mcm6>2 ATP	Mcm2>5 ATP	Mcm5>3 ATP	Mcm3>7 ADP	Mcm7>4 ADP
R finger β Ca > WA K β Ca (Å)	15.0	13.3	12.7	13.8	14.9	15.4
R finger β Ca > S3 H β Ca (Å)	14.0	14.3	13.7	13.8	14.2	14.3
Inter-AAA+ surface ($Å^2$)	1410	1626	1872	1892	1242	867
Inter-active site surface ($Å^2$)	129	181	195	195	118.6	112

STATE 2A	Mcm4>6 ATP	Mcm6>2 ATP	Mcm2>5 ADP	Mcm5>3 ATP	Mcm3>7 ADP	Mcm7>4 ATP
R finger β Ca > WA K β Ca (Å)	14.6	13.4	16.8	12.6	14.7	14.0
R finger β Ca > S3 H β Ca (Å)	11.3	10.0	17.5	11.3	14.2	13.0
Inter-AAA+ surface ($Å^2$)	1706	1437	1264	1421	1309	1785
Inter-active site surface ($Å^2$)	190	148	144	138	128	158

STATE 2B	Mcm4>6 ATP	Mcm6>2 ADP	Mcm2>5 ADP	Mcm5>3 ATP	Mcm3>7 ATP	Mcm7>4 ATP
R finger β Ca > WA K β Ca (Å)	15.5	15.0	13.3	12.4	13.77	14.0
R finger β Ca > S3 H β Ca (Å)	13.0	14.3	15.7	12.0	13.0	13.8
Inter-AAA+ surface ($Å^2$)	1684	900	1866	1423	1603	1746
Inter-active site surface ($Å^2$)	168	155	117	166	228	122

Table S2 related to Figure 4**:** ATPase interface measurements, including *i.* distance between Arg finger beta carbon and Walker A Lys beta carbon, *ii.* distance between Arg finger beta carbon and Sensor 3 His beta carbon, *iii.* inter-protomer interface area, measured between neighbouring AAA+ modules and *iv.* Inter-protomer interface area limited to active site residues surrounding the nucleotide.

A: 5’-GGCAGGCAGGCAGGCAGGCAGGCAGGCAGGCAGGCAGGCAGGTTGGCGAATTC-3’
B: [5’-Phos]CCATTGCCTCAGCATCCGGTACCTCAGCACGACGTTGTAAAACGAGC[3’-Cy5]
C: 5’-GGCTCGTTTTACAACGTCGTGCTGAGGTAC[5FdC]GGATGCTGAGGCAATGGGAATTCGCCAACCTTTTTTTTTTTTTTTTTTTTTTTTTTTTTTTTTTTTTTTTTTTTT-3’
D: 5’-GGCAGGCAGGCAGGCAGGCAGGCAGGCAGGCAGGCAGGCAGGTTGGCGAATTCCCATTGCCTCAGCATC[5FdC]GGTACCTCAGCACGACGTTGTAAAACGAG-3’
E: [5’-Cy5]GCTCGTTTTACAACGTCGTGCTGAGGTACCGGATGCTGAGGCAATGGGAATTCGCCAACCTTTTTTTTTTTTTTTTTTTTTTTTTTTTTTTTTTTTTTTTTTTTT-3’
F: [5’-Phos]TGAGGCAATGGGAATTCGCCAACCTTTTTTTTTTTTTTTTTTTTTTTTTTTTTTTTTTTTTTTTTT-3’
G: [5’-Desthiobiotin]GCTCGTTTTACAACGTCGTGC-3’
H: [5’-Phos]TGAGGTAC[5-fluoro-2’-dC]GGATGC-3’
I: 5’-GCCAGCCAGCCAGCCAGCCAGCCAGCCAGCCAGCCAGCCAGGTTGGCGAATTCCCATTGCCTCAGCATCCGGTACCTCAGCATCCGGTACCTCAGCACGACGTTGTAAAACGAGC-3’
J: 5’-GCTCGTTTTACTACGTCGTGCTGAGGTAC[5FdC]GGATGCTCTCAGACCGGACA-3’
K: [5’-Phos]ATCCGGTACCTCAGCACGACGTAGTAAAACGAGC[3’-Desthiobiotin]
L: 5’-GGCAGGCAGGCAGGCAGGCAGGCAGGCAGGCAGGCAGGCAGGTTGGCGAATTCCCATTGCCTCATGTC[5-fluoro-2’-dC]GGTCTGAGAGC-3’
M: [5’-Phos]TGAGGCAATGGGAATTCGCCAACCTTTTTTTTTTTTTTTTTTTTTTTTTTTTTTTTTTTTTTTTTT-3’
N: [5'-Desthiobiotin]GCAGCCACGCTGGCCGTTTTACAACGTCGTGACTGGGCACTTGATCGGCCAACCTTTTTTTTTT TTTTTTTTTTTTTTTTTTTTTTTTTTTTTTTTT-3'
O: 5'-GGCAGGCAGGCAGGCAGGCAGGCCGTGCGCGTGGTCGTGCGGTTGGCCGATCAAGTGCCCAGTCACGACGTTGTAAAACGGCCAGCGTGGCTGC-3'

P: 5’-ATATATCCGGCCTGTATTTTACAGATTTTATGTTTAGATCTTTTATGCTTGCTTTTCAAAAGGCCTGCAGGCAAGTGCACAAACAATACTTAAATAAATACTACTCAGTAATAACCTATTTCTTAGCATTTTTGACGAAATTTGCTATTTTCCCAGTTCCGGCGCCCA-3’
Q: 5’-AGGCAATGGGAATTCGCCAACCT-3’
R: [5’-Desthiobiotin]ATATATCCGGCCTGTATTTTACAGATTTTATGTTTAGATCTTTTATGC-3’
S: 5'-AGGGCGCCGGAACTGGGAAAATAGCAAATTTCGTCAAAAATGC-3’
T: 5'-TTTTTTTTTTTTTTTTTTTTTTTTTTTTTTGGTTTTCCCAGTCACGACGTTGTAAAACGACGGCCAGTGC-3'

Table S3 related to STAR Methods**:** List of oligonucleotides.
